# Residential proximity to croplands at birth and childhood leukaemia

**DOI:** 10.1186/s12940-022-00909-0

**Published:** 2022-10-27

**Authors:** Sophie Bamouni, Denis Hémon, Laure Faure, Jacqueline Clavel, Stéphanie Goujon

**Affiliations:** 1 Inserm, UMR 1153 Center of Research in Epidemiology and StatisticS (CRESS), Epidemiology of Childhood and Adolescent Cancers Team (EPICEA), 16 avenue Paul Vaillant Couturier, Hôpital Paul Brousse – Bât Leriche/porte 45, F-94807 Villejuif Cedex, France; 2 Université Paris Cité, Paris, France; 3 French National Registry of Childhood Haematological Malignancies (RNHE), F-94807 Villejuif, France

**Keywords:** Childhood leukaemia, Agricultural crops, Agricultural census, Pesticides, Prenatal exposure

## Abstract

**Background:**

Domestic and parental occupational pesticide exposures are suspected of involvement in the occurrence of childhood acute leukaemia (AL), but the role of exposure to agricultural activities is little known. In a previous ecological study conducted in France, we observed an increase in acute lymphoblastic leukaemia (ALL) incidence rate with increasing viticulture density in the municipalities of residence at diagnosis.

**Objectives:**

This study aimed to test the hypothesis that residential proximity to croplands at birth increases the risk of childhood AL, with a particular focus on vineyards.

**Methods:**

We identified all the primary AL cases diagnosed before the age of 15 years in the cohorts of children born in the French municipalities between 1990 and 2015. We estimated crop densities in each municipality of residence at birth using agricultural census data, for ten crop types. Variations in standardized incidence ratios (SIR) were evaluated with Poisson regression models, for all AL, ALL and acute myeloid leukaemia (AML), separately.

**Results:**

Among the 19,809,700 children born and residing in mainland France at birth in 1990–2015, 8,747 AL cases (7,236 ALL and 1,335 AML) were diagnosed over the period. We did not evidence any statistically significant positive association between total crop density or any specific crop density in the municipality of residence at birth and all AL, ALL or AML. Interestingly, we observed a higher ALL incidence rate in the municipalities with the highest viticulture densities (SIR = 1.25 95%CI [1.01–1.54]). Adjusting for the main potential confounders did not change the results.

**Conclusion:**

Our study does not support the hypothesis that residential proximity to croplands, particularly vineyards, around birth plays a role in childhood leukaemia. The slightly higher ALL incidence rate in children born in the municipalities with the highest viticulture densities may reflect the previously-observed association at diagnosis.

**Supplementary Information:**

The online version contains supplementary material available at 10.1186/s12940-022-00909-0.

## Introduction

Acute leukaemia (AL), the most frequent childhood cancer, affects around 500 children under 15 each year in France [1]. About 80% of the cases consist in acute lymphoblastic leukaemia (ALL) and 15% in acute myeloid leukaemia (AML). Domestic pesticide use and parental occupational exposure to pesticides are strongly suspected of playing a role in childhood AL. A positive association between childhood AL risk and domestic pesticide use during pregnancy and childhood has been reported in several studies and confirmed by recent meta-analyses [2–4]. Pooled-analysis meta-analyses by the CLIC consortium have shown associations between maternal occupational exposure during pregnancy and AML risk, and between paternal occupational exposure preconception and childhood ALL [5]. Proximity to cropland and agricultural pesticide exposure have been subject to less investigation. Several case–control studies have exploited land-use data [6, 7], satellite images [8] or aerial photographs [9] to estimate the agricultural area in the vicinity of the participants’ geocoded residential addresses, while others, with an ecological design, considered agricultural census data to evaluate the agricultural activity in the area of residence (county or municipality level) [10–13]. Some studies reported positive associations with particular crop types, with, however, no consistency [7, 8, 10, 11]. In California, where agricultural pesticide applications are systematically registered with information on the type of pesticides used, quantity applied, crop type and treated area, some studies [14–17] found a greater AL risk with several chemical types of pesticide, while in another study no association was observed [18]. In Denmark, a cohort study estimated agricultural pesticide exposure around the geocoded addresses of residence during pregnancy based on pesticide sales data and did not identify any association with childhood AL risk [19].

In a recent ecological study, we observed a log-linear increase in ALL incidence rate with increasing viticulture density in the municipalities of residence at the time of diagnosis, with a 16% increase in the municipalities in which more than 25% of their area was devoted to viticulture [12].

In this study, we investigated whether the previously-observed association with viticulture near the residence at the time of diagnosis might reflect a stronger relationship with prenatal exposure. More generally, we tested the hypothesis that the proximity of home to crops and agricultural activities during the prenatal period, a critical period for leukaemogenesis, increased the risk of childhood AL. The study was conducted on the population of children born between 1990 and 2015 in mainland France and took the place of residence at birth as a proxy for residence during the prenatal period.

## Materials and methods

### Population and incidence data

The population under study consisted in children under 15 born in mainland France between 1990 and 2015. The 19,809,700 births (761,873 per year on average) domiciled in the municipalities of mainland France over the study period were provided by the French National Institute for Statistics and Economic Studies (INSEE). The at-risk populations were estimated assuming that death and migration rates were negligible.

All the primary AL cases in that population were identified in the French national registry of childhood cancer (RNCE). Of the 9,293 AL cases born and diagnosed in 1990–2015, 269 cases born abroad were excluded. We obtained legal authorisation to access the birth certificates from the town halls of the municipalities of birth (i.e. the municipalities where the maternity hospitals were located) that were recorded in the RNCE. Birth certificates stating the address of the mothers' residences were obtained for 97% of the remaining cases (277 missing addresses). A total of 8,747 AL cases aged under 15 were identified among the children born in 1990–2015 and domiciled at birth in one of the 35,511 municipalities of mainland France. There were 7,236 ALL (6,970 B-cell precursor ALL, BCP-ALL), and 1,335 AML cases.

### Crop density

The crop-growing areas of the municipalities were estimated for the birth periods 1990–1994, 1995–2004, and 2005–2015 using data from the 1988, 2000, and 2010 agricultural censuses, respectively [20]. Crop density was defined as the ratio of the crop area to the total municipality area (about 15 km^2^ on average) for each permanent, i.e. viticulture and arboriculture, and non-permanent crop, i.e. straw cereals, maize, rapeseed, sunflowers, fresh vegetables, dry vegetables and protein crops, potatoes, and beet.

### Statistical analysis

We used Poisson regression models to test the association between municipality crop densities and AL incidence rates. The number of AL cases expected over the study period was estimated for each birth period (1990–1994, 1995–2004, 2005–2015) and each municipality of residence at birth using national age-specific incidence rates as reference rates. Age was considered in one-year categories for AL and ALL, and grouped into four categories (< 1, 1–4, 5–9, 10–14) for AML. We checked that the annual 0–14-year and age-specific incidence rates were homogeneous over the period 1990–2015 (Additional Fig. 1).

Total crop density was first considered as a 5-category variable in which the first category was constituted by municipalities with a total crop density of less than 5%, and the others by the population-weighted quartiles of the total crop density [12].

For crop-specific densities, we added a category for the municipalities with at least 5% of total crops but less than 5% of the specific crop.

For additional analyses on viticulture densities, we split the quartiles of density into semi-quartiles to better describe the right tail of their distribution.

The standardized incidence ratios (SIR) between observed (O) and expected (E) numbers of AL cases, and their Wald-based 95% confidence intervals, were estimated for each density category using the following Poisson regression model:

1 $$ln\left(E\left({o}_{m,p}/X\right)\right)=ln\left({E}_{m,p}\right)+{\Sigma }_{k}{\updelta }_{k}\times {\mathrm{X}}_{k}\left(m\right)$$

in which O_*m,p*_ is the number of cases for the municipality *m* and the birth period *p;* ln(E_*m,p*_) is the offset*; X*_*k*_(*m*) is a dummy variable equal to one if municipality *m* belongs to the kth crop density category during the birth period *p*, 0 otherwise (k = 1 to 5 for the total crop density, k = 1 to 6 for a specific crop density); *δ*_*k*_ is the parameter associated with the kth crop density category so that exp(*δ*_*k*_) corresponds to the SIR for this category. We used a likelihood ratio test that compared model [1] to the null model (only an intercept included) to test for heterogeneity between SIRs of the crop density categories.

In a quantitative approach, the SIR variations (SIRR) for a 10% increase in crop density were also estimated considering crop densities as continuous variables. The log-linearity hypothesis was first tested by comparing the fits of the qualitative model [1] and the following semi-quantitative model [2] with a likelihood ratio test.

2 $$ln\left(E\left({o}_{m,p}/Y\right)\right)=ln\left({E}_{m,p}\right)+\mathrm{\alpha }+\upbeta \times {Y}_{k(m)}$$

In which Y_k(m)_ is the population-weighted average value of the crop density in the kth category to which the municipality *m* belongs during the period *p*, and *β* is the slope parameter.

When the log-linearity hypothesis was not rejected, we fitted a model including crop density as a continuous variable:

3 $$ln\left(E\left({o}_{m,p}/Y\right)\right)=ln\left({E}_{m,p}\right)+{\mathrm{\alpha }}^{^{\prime}}+{\upbeta }^{^{\prime}}\times {Y}_{m}$$

In which Y_*m*_ is the crop density in the municipality *m* during the period *p* and β’ is the slope parameter. In this study, Y was calibrated so that exp ($\widehat{\beta}^{\prime}$) estimated the SIRR for an increase of 10% in the crop density of the municipality.

The size of urban unit (Paris urban unit *vs*. other urban units) was systematically included in the exposure–response models for AL, ALL and BCP-ALL in order to improve model fit.

As AL incidence rates were not spatially correlated on the municipality scale [21], we did not include a spatial autocorrelation term in the models.

The statistical significance level was set at 5% for all the tests and the p-values for exposure–response models were estimated using one-sided tests.

The analyses were conducted for all AL taken together, and separately for ALL (particularly BCP-ALL) and AML. Total crop and specific crop densities were analyzed separately.

### Sensitivity analysis

The size of the urban unit (Paris urban unit *vs*. other urban units), residential UV radiation exposure (UV > 105.5 J/cm^2^*vs*. UV ≤ 105.5 J/cm^2^) and French deprivation index FDep (Rey et al., 2009) (the most deprived quintile Q5 *vs.* other quintiles Q1-Q4), which were associated with childhood ALL in our previous studies [22, 23], were considered as potential confounders and adjusted for in the sensitivity analyses conducted on ALL and BCP-ALL. Additional analyses were performed after excluding the Paris urban unit or urban units with a population greater than 100,000, and by stratifying the analyses by age group (0–6 and 7–14 years old). For viticulture, we also performed an analysis for ALL restricted to the 1990–2004 birth period in order to account for a vine uprooting policy introduced in 2007.

All the analyses were performed using R 3.6.3 software (https://cran.r-project.org/).

## Results

**Fig. 1:**
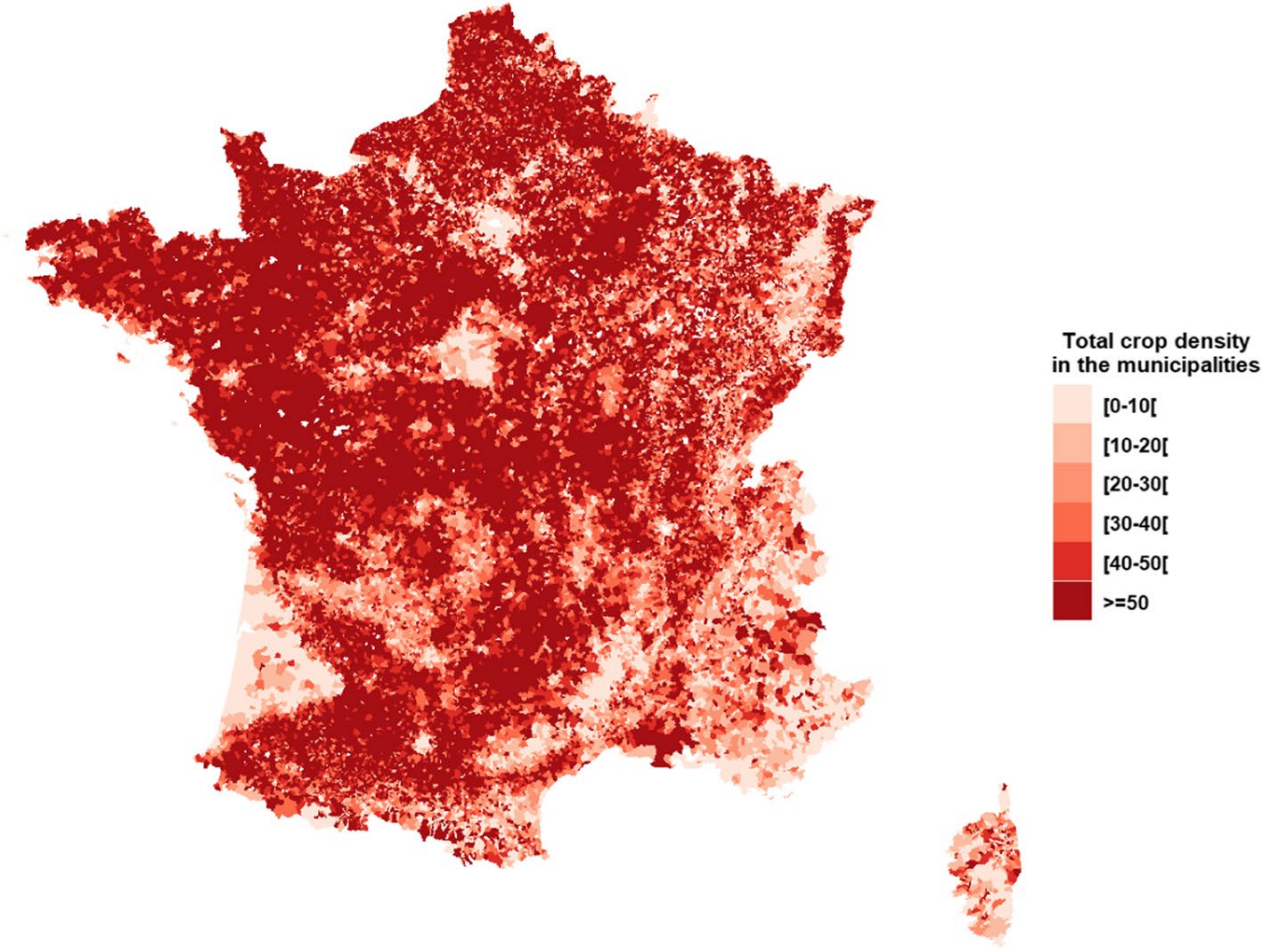
Total crop density (%) in the French municipalities – Agricultural census 2010

**Fig. 2:**
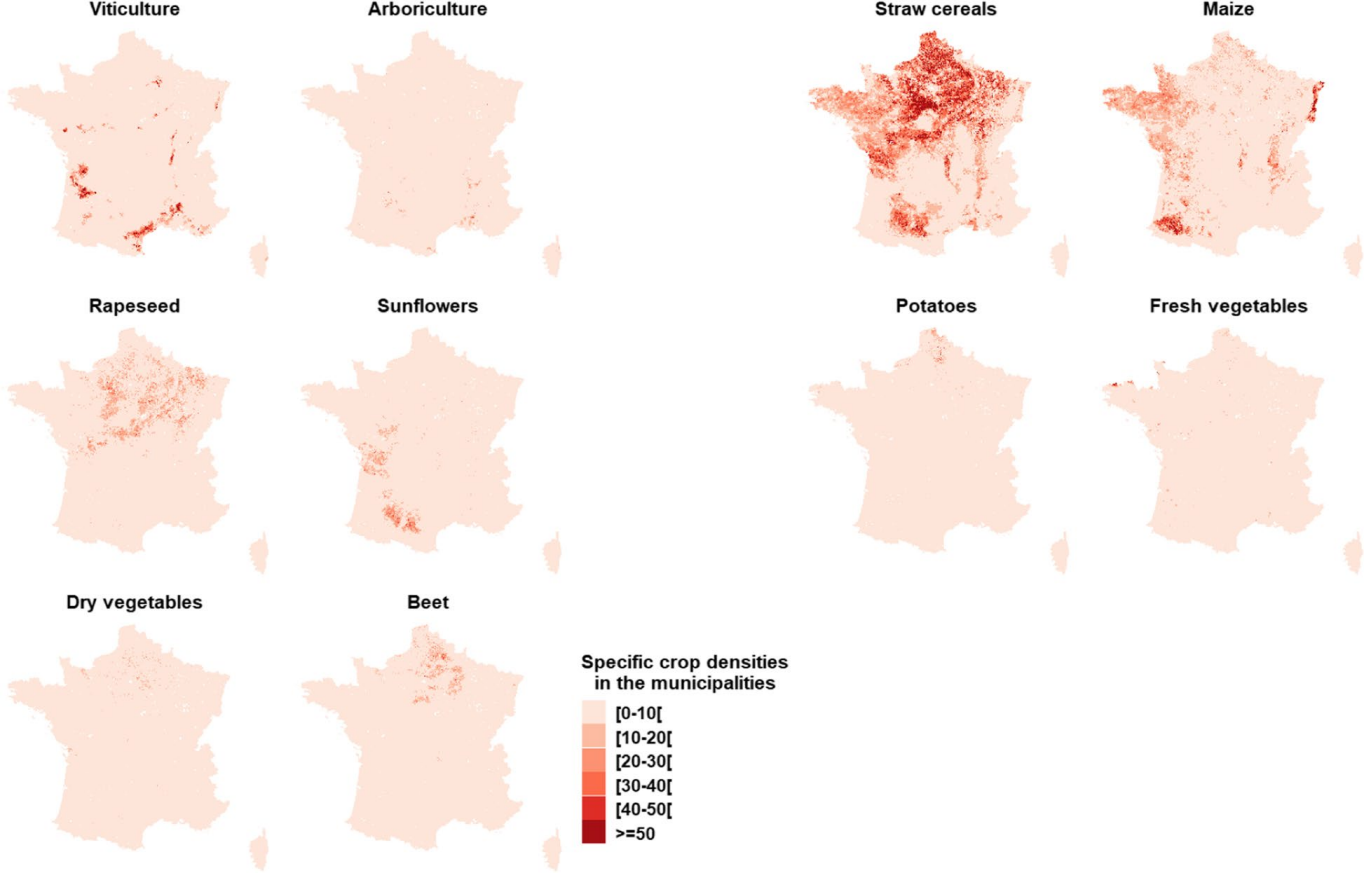
Specific crop densities (%) in the French municipalities – Agricultural census 2010

One third of the births were domiciled in 5.7% of the municipalities with a total crop density lower than 5%. The majority of municipalities had at least 50% cropland (Fig. 1). The distribution of total crop and specific crop densities weighted by the number of births in the municipalities are shown in Table 1. The municipalities with at least 19% total crop area accounted for 50% of births over the study period. The crop specific distributions were asymmetric and varied depending on the type of crop. In particular, for viticulture, very few children (1%) lived at the time of birth in municipalities with a high viticulture density (p99 = 33.1%). The spatial distribution of specific crop densities is shown in Fig. 2. Intensive viticulture was mainly practiced in the South-West and Mediterranean regions. Arboriculture is quite rare in France. Straw cereals are the most common type of crop and predominant in the North of France.

**Table 1:** Distribution^a^ of the total crop and specific crop densities in the French municipalities

Crop	P5	P25	P50	P75	P95	P99
***All crops***	0	1.5	19.0	48.1	73.5	115.7^b^
***Permanent crops***						
Viticulture	0	0	0	0.03	1.5	33.1
Arboriculture	0	0	0	0.1	0.9	11.0
***Non-permanent crops***						
Straw cereals	0	0	2.6	11.8	24.3	55.8
Fresh vegetables	0	0	0.02	0.3	1.5	9.2
Maize	0	0	0	2.1	7.6	21.8
Rapeseed	0	0	0	0.8	3.4	13.1
Sunflower	0	0	0	0	1.7	11.9
Potatoes	0	0	0	0.04	0.4	7.9
Dry vegetables	0	0	0	0.2	1.7	8.8
Beet	0	0	0	0	1.6	13.8

P5, P25, P50, P75, P95, and P99: 5th, 25th, 50th, 75th, 95th and 99th population weighted percentiles of the crop densities (%), respectively

^a^Distribution weighted by numbers of births in the municipalities, over 3 birth periods (based on the 1988, 2000 and 2010 agricultural censuses)

^b^99^th^ percentile is greater than 100% because the cropland areas reported in the agricultural censuses are assigned to the municipality of the farm headquarter, which may not be the municipality where the land plots are situated

We did not evidence any association between total crop density in the municipality of residence at birth and all AL, ALL and AML (Table 2).

**Table 2:** Association between childhood leukaemia incidence rate and crop densities^a^ (all crops and permanent crops)

	**AL (*N* = 8 747)**					**ALL (*N* = 7 236)**					**AML (*N* = 1 335)**				
	**O**	**E**	**SIR**	**95% CI**	**p**^**b**^	**O**	**E**	**SIR**	**95% CI**	**p**^**b**^	**O**	**E**	**SIR**	**95% CI**	**p**^**b**^
**Total crops**															
Total crop density < 5%	2758	2788.8	0.99	0.95–1.03	0.54	2285	2308.5	0.99	0.95–1.03	0.67	422	440.7	0.96	0.87–1.05	0.26
[5–18.7]	1476	1485.4	0.99	0.94–1.05		1211	1228.4	0.99	0.93–1.04		231	224.1	1.03	0.91–1.17	
[18.7–37.4]	1507	1485.1	1.01	0.96–1.07		1263	1228.2	1.03	0.97–1.09		212	223.3	0.95	0.83–1.09	
[37.4–61.0]	1541	1490.7	1.03	0.98–1.09		1259	1232.8	1.02	0.97–1.08		252	223.3	1.13	1.00–1.28	
≥ 61	1465	1497.1	0.98	0.93–1.03		1218	1238.1	0.98	0.93–1.04		218	223.6	0.98	0.85–1.11	
Test of departure from log-linearity					0.38					0.51					0.18
SIRR for ∆x = 10%^c^			1.00	0.99–1.01	0.47		1238.1	1.00	0.99–1.01	0.44		223.6	1.00	0.99–1.01	0.31
**Viticulture**															
Total crop density < 5%	2758	2788.8	0.99	0.95–1.03	0.84	2285	2308.5	0.99	0.95–1.03	0.88	422	440.7	0.96	0.87–1.05	0.62
Total crop density ≥ 5% and viticulture < 5%	5413	5391.7	1.00	0.98–1.03		4465	4459.2	1.00	0.97–1.03		834	809.7	1.03	0.96–1.10	
[5–10.0]	143	141.2	1.01	0.86–1.19		124	116.8	1.06	0.89–1.27		17	21.1	0.81	0.50–1.30	
[10.0–16.2]	141	143.7	0.98	0.83–1.16		116	118.8	0.98	0.81–1.17		22	21.5	1.02	0.67–1.56	
[16.2–24.7]	138	142.0	0.97	0.82–1.15		120	117.5	1.02	0.85–1.22		17	21.2	0.80	0.50–1.29	
≥ 24.7	154	139.5	1.10	0.94–1.29		126	115.4	1.09	0.92–1.30		23	20.8	1.10	0.73–1.66	
Test of departure from log-linearity					0.48					0.94					0.48
SIRR for ∆x = 10%^c^			1.01	0.98–1.04	0.24		115.4	1.01	0.98–1.05	0.23		20.8	1.01	0.93–1.09	0.45
**Arboriculture**															
Total crop density < 5%	2758	2788.8	0.99	0.95–1.03	0.07	2285	2308.5	0.99	0.95–1.03	0.15	422	440.7	0.96	0.87–1.05	0.33
Total crop density ≥ 5% and arboriculture < 5%	5809	5742.1	1.01	0.99–1.04		4799	4748.8	1.01	0.98–1.04		887	861.8	1.03	0.96–1.10	
[5–6.4[	54	54.1	1.00	0.76–1.30		45	44.7	1.01	0.75–1.35		9	8.2	1.10	0.57–2.12	
[6.4–9.34[	38	53.3	0.71	0.52–0.98		30	44.1	0.68	0.48–0.97		7	8.1	0.87	0.41–1.82	
[9.4–13.1[	42	51.1	0.82	0.61–1.11		39	42.2	0.92	0.67–1.26		3	7.6	0.39	0.13–1.22	
≥ 13.1	46	57.6	0.80	0.60–1.07		38	47.6	0.80	0.58–1.10		7	8.6	0.81	0.39–1.70	
Test of departure from log-linearity					0.36					0.38					0.35
SIRR for ∆x = 10%^c^			0.87	0.79–0.97	0.99		47.6	0.89	0.79–0.99	0.98		8.6	0.78	0.58–1.06	0.94

*AL* Acute leukaemia, *ALL* Acute lymphoblastic leukaemia, *AML* Acute myeloid leukaemia, *N* Number of cases, *O* Observed number of cases, *E* Expected number of cases, *SIR* Standardized incidence ratio, 95% CI 95% Confidence interval,

^a^ The total crop density and the specific crop density in a municipality were defined as the ratio of the total area used for agriculture and the area used for the specific crop, respectively, over the total area of the municipality (based on national agricultural census data). Separate models were used for each specific crop as well as for total crops

^b^ p-value of the tests (chi-square test of heterogeneity between SIRs in categories of crop density, test for departure from the log-linearity hypothesis and, test for the slope parameter in the linear Poisson regression model, H0: β ≤ 0 *vs* H1: β > 0)

^c^ SIRR (for Δ x = 10%) = Relative Standardized Incidence Ratio: multiplicative variation in the SIR for a 10% increase in the crop density derived from a linear Poisson regression model, with adjustment on the size of urban unit (Paris vs other urban units) for AL and ALL

For viticulture, no statistically significant heterogeneity of the SIRs for the density categories was observed for AL or its subgroups; there was no evidence of a log-linear association (Table 2). The SIR was slightly higher in the municipalities with the highest viticulture densities (1.10 95%CI [0.94–1.29] for AL) while closer to 1 for the other categories. Although far from being statistically significant, the slight increase for the highest category resembles that observed in our ecological study at the time of diagnosis.

**Fig. 3:**
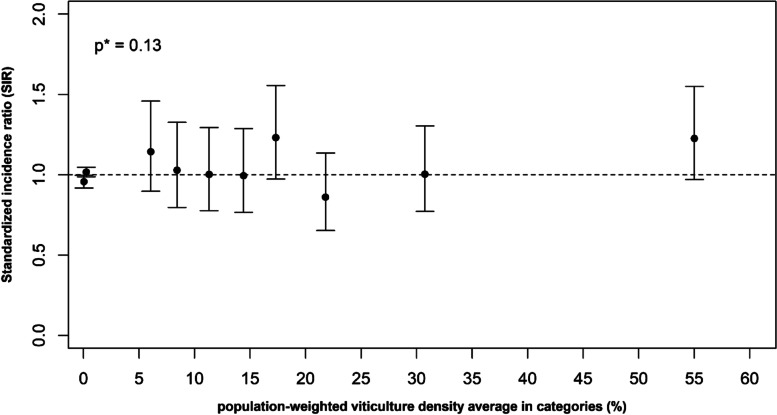
Standardized incidence ratio of acute lymphoblastic leukaemia (ALL) in viticulture density^a^ categories based on population-weighted semi-quartiles. Legend: ^a^ The viticulture density in a municipality is defined as the ratio of the total area used for viticulture over the total area of this municipality. The categories were defined as follows: 1^st^ category: municipalities with a total crop density < 5%, 2^nd^ category: municipalities with viticulture density < 5%, 3^rd^ to 10.^th^ categories: 8 groups of municipalities with viticulture density ≥ 5% and equal number of births over the study period (1990–2015). *p-value of the chi-square test of heterogeneity of the viticulture density categories

We subdivided the viticulture density quartiles into semi-quartiles. There was no change in the log-linear trend parameter estimates, but a greater SIR in the highest density category (municipalities with a viticulture density greater than 37.7%) was detected for AL and ALL (SIR = 1.25 95%CI [1.01–1.54] and SIR = 1.23 95%CI [0.97–1.55], respectively). The SIRs for ALL are presented in Fig. 3.

The arboriculture density analyses did not show any positive association with AL or its subgroups (Table 2).

We did not observe any heterogeneity of the SIRs for the density categories of non-permanent crops, or any positive log-linear association with AL or its subgroups, ALL and AML (Table 3).

**Table 3:** Association between the incidence rate of childhood leukaemia and non-permanent crop densities^a^

	**AL (*N* = 8 747)**					**ALL (*N* = 7 236)**					**AML (*N* = 1 335)**				
	**O**	**E**	**SIR**	**95% CI**	**p**^**b**^	**O**	**E**	**SIR**	**95% CI**	**p**^**b**^	**O**	**E**	**SIR**	**95% CI**	**p**^**b**^
**Straw cereals**															
Total crop density < 5%	2758	2788.8	0.99	0.95–1.03	0.87	2285	2308.5	0.99	0.95–1.03	0.69	422	440.7	0.96	0.87–1.05	0.84
Total crop density ≥ 5% and straw cereals < 5%	2271	2290.1	0.99	0.95–1.03		1868	1893.8	0.99	0.94–1.03		346	343.5	1.01	0.91–1.12	
[5.0–8.8]	920	912.7	1.01	0.94–1.08		759	754.9	1.01	0.94–1.08		143	137.1	1.04	0.89–1.23	
[8.8–14.2]	944	910.5	1.04	0.97–1.11		796	753.1	1.06	0.99–1.13		132	136.7	0.97	0.81–1.14	
[14.2–23.8]	920	919.4	1.00	0.94–1.07		758	760.4	1.00	0.93–1.07		146	138.2	1.06	0.90–1.24	
≥ 23.8	934	925.4	1.01	0.95–1.08		770	765.3	1.01	0.94–1.08		146	138.7	1.05	0.89–1.24	
Test of departure from log-linearity					0.84					0.61					0.89
SIRR for ∆x = 10%^c^			1.00	0.99–1.02	0.36		765.3	1.00	0.98–1.02	0.42		138.7	1.02	0.98–1.06	0.14
**Maize**															
Total crop density < 5%	2758	2788.8	0.99	0.95–1.03	0.39	2285	2308.5	0.99	0.95–1.03	0.73	422	440.7	0.96	0.87–1.05	0.25
Total crop density ≥ 5% and maize < 5%	4666	4612.2	1.01	0.98–1.04		3850	3815.0	1.01	0.98–1.04		716	693.3	1.03	0.96–1.11	
[5–7.0]	314	332.9	0.94	0.84–1.05		273	275.2	0.99	0.88–1.12		37	49.8	0.74	0.54–1.03	
[7.03–9.8]	345	332.4	1.04	0.93–1.15		286	274.8	1.04	0.93–1.17		53	49.6	1.07	0.82–1.40	
[9.83–14.4]	347	335.8	1.03	0.93–1.15		279	277.6	1.01	0.89–1.13		58	50.1	1.16	0.89–1.50	
≥ 14.4	317	344.9	0.92	0.82–1.03		263	285.0	0.92	0.82–1.04		49	51.5	0.95	0.72–1.26	
Test of departure from log-linearity					0.38					0.75					0.16
SIRR for ∆x = 10%^c^			1.00	0.95–1.04	0.59		285.0	0.99	0.95–1.04	0.60		51.50	1.04	0.93–1.15	0.25
**Rapeseed**															
Total crop density < 5%	2758	2788.8	0.99	0.95–1.03	0.79	2285	2308.5	0.99	0.95–1.03	0.65	422	440.7	0.96	0.87–1.05	0.47
Total crop density ≥ 5% and rapeseed < 5%	5392	5388.0	1.00	0.97–1.03		4450	4456.1	1.00	0.97–1.03		828	808.6	1.02	0.96–1.10	
[5–6.1]	153	140.3	1.09	0.93–1.28		129	116.0	1.11	0.94–1.32		24	21.2	1.13	0.76–1.69	
[6.1–7.9]	152	140.8	1.08	0.92–1.27		130	116.4	1.12	0.94–1.33		18	21.2	0.85	0.54–1.35	
[7.9–11.0]	141	141.4	1.00	0.85–1.18		121	116.9	1.03	0.87–1.24		16	21.2	0.75	0.46–1.23	
≥ 11.0	151	147.7	1.02	0.87–1.20		121	122.1	0.99	0.83–1.18		27	22.1	1.22	0.84–1.78	
Test of departure from log-linearity					0.79					0.62					0.36
SIRR for ∆x = 10%^c^			1.02	0.95–1.10	0.30		122.1	1.03	0.95–1.11	0.26		22.10	1.01	0.83–1.22	0.46
**Sunflowers**															
Total crop density < 5%	2758	2788.8	0.99	0.95–1.03	0.62	2285	2308.5	0.99	0.95–1.03	0.72	422	440.7	0.96	0.87–1.05	0.48
Total crop density ≥ 5% and sunflowers < 5%	5634	5600.6	1.01	0.98–1.03		4656	4631.8	1.01	0.98–1.03		853	840.9	1.01	0.95–1.08	
[5–6.2]	75	88.9	0.84	0.67–1.06		64	73.6	0.87	0.68–1.11		11	13.3	0.83	0.46–1.50	
[6.2–8.2]	91	88.9	1.02	0.83–1.26		71	73.6	0.97	0.76–1.22		20	13.3	1.51	0.97–2.33	
[8.2–11.9]	92	89.9	1.02	0.83–1.26		77	74.4	1.04	0.83–1.29		15	13.4	1.12	0.67–1.85	
≥ 11.9	97	89.8	1.08	0.89–1.32		83	74.3	1.12	0.90–1.39		14	13.4	1.04	0.62–1.76	
Test of departure from log-linearity					0.51					0.62					0.42
SIRR for ∆x = 10%			1.00	0.91–1.09	0.53		74.3	1.01	0.92–1.11	0.43		13.41	1.06	0.86–1.32	0.28
**Potatoes**															
Total crop density < 5%	2758	2788.8	0.99	0.95–1.03	0.73	2285	2308.5	0.99	0.95–1.03	0.68	422	440.7	0.96	0.87–1.05	0.42
Total crop density ≥ 5% and potatoes < 5%	5803	5792.4	1.00	0.98–1.03		4798	4790.5	1.00	0.97–1.03		885	869.6	1.02	0.95–1.09	
[5–6.3]	46	41.1	1.12	0.84–1.49		42	34.0	1.24	0.91–1.67		4	6.1	0.65	0.24–1.74	
[6.3–8.5]	47	41.7	1.13	0.85–1.50		34	34.5	0.99	0.70–1.38		11	6.2	1.77	0.98–3.19	
[8.47–12.6]	48	41.6	1.15	0.87–1.53		40	34.4	1.16	0.85–1.58		7	6.2	1.13	0.54–2.36	
≥ 12.6	45	41.3	1.09	0.81–1.46		37	34.2	1.08	0.78–1.50		6	6.2	0.97	0.44–2.17	
Test of departure from log-linearity					0.92					0.77					0.32
SIRR for ∆x = 10%^c^			1.06	0.95–1.20	0.15		34.2	1.04	0.91–1.18	0.30		6.16	1.13	0.85–1.51	0.20
**Fresh vegetables**															
Total crop density < 5%	2758	2788.8	0.99	0.95–1.03	0.10	2285	2308.5	0.99	0.95–1.03	0.34	422	440.7	0.96	0.87–1.05	0.50
Total crop density ≥ 5% and fresh vegetables < 5%	5758	5729.6	1.00	0.98–1.03		4763	4738.5	1.01	0.98–1.03		877	859.9	1.02	0.95–1.09	
[5–6.11]	62	56.8	1.09	0.85–1.40		52	47.0	1.11	0.84–1.45		9	8.6	1.04	0.54–2.00	
[6.11–7.7]	40	57.1	0.70	0.51–0.95		35	47.3	0.74	0.53–1.03		5	8.5	0.59	0.24–1.41	
[7.7–11.5]	58	56.4	1.03	0.80–1.33		45	46.6	0.97	0.72–1.29		12	8.5	1.42	0.81–2.50	
≥ 11.5	71	58.3	1.22	0.97–1.54		56	48.2	1.16	0.89–1.51		10	8.8	1.14	0.61–2.12	
Test of departure from log-linearity					0.08					0.26					0.40
SIRR for ∆x = 10%^c^			1.07	0.98–1.18	0.05		48.2	1.04	0.94–1.16	0.20		8.79	1.18	0.96–1.44	0.05
**Dry vegetables**															
Total crop density < 5%	2758	2788.8	0.99	0.95–1.03	0.75	2285	2308.5	0.99	0.95–1.03	0.64	422	440.7	0.96	0.87–1.05	0.58
Total crop density ≥ 5% and dry vegetables < 5%	5725	5687.1	1.01	0.98–1.03		4740	4703.3	1.01	0.98–1.04		864	853.7	1.01	0.95–1.08	
[5–5.9]	63	67.7	0.93	0.73–1.19		47	56.0	0.84	0.63–1.12		15	10.1	1.48	0.89–2.46	
[5.9–7.2]	59	67.7	0.87	0.68–1.13		48	56.0	0.86	0.65–1.14		11	10.1	1.08	0.60–1.96	
[7.23–9.8]	68	68.0	1.00	0.79–1.27		57	56.3	1.01	0.78–1.31		10	10.2	0.98	0.53–1.82	
≥ 9.8	74	67.7	1.09	0.87–1.37		59	56.0	1.05	0.82–1.36		13	10.1	1.29	0.75–2.21	
Test of departure from log-linearity					0.62					0.50					0.72
SIRR for ∆x = 10%^c^			0.95	0.84–1.06	0.83		56.0	0.89	0.78–1.02	0.95		10.11	1.14	0.96–1.36	0.07
**Beet**															
Total crop density < 5%	2758	2788.8	0.99	0.95–1.03	0.60	2285	2308.5	0.99	0.95–1.03	0.76	422	440.7	0.96	0.87–1.05	0.49
Total crop density ≥ 5% and beets < 5%	5601	5545.5	1.01	0.98–1.04		4631	4586.2	1.01	0.98–1.04		854	832.3	1.03	0.96–1.10	
[5–6.9]	91	103.9	0.88	0.71–1.08		75	85.9	0.87	0.70–1.09		12	15.6	0.77	0.44–1.36	
[6.9–9.0]	95	103.4	0.92	0.75–1.12		82	85.5	0.96	0.77–1.19		12	15.6	0.77	0.44–1.36	
[9.04–13.0]	105	102.7	1.02	0.84–1.24		83	84.9	0.98	0.79–1.21		20	15.4	1.30	0.84–2.02	
≥ 13	97	102.6	0.95	0.77–1.15		80	84.9	0.94	0.76–1.17		15	15.5	0.97	0.59–1.61	
Test of departure from log-linearity					0.59					0.78					0.35
SIRR for ∆x = 10%^c^			0.95	0.87–1.03	0.90		84.9	0.94	0.86–1.03	0.90		15.45	1.01	0.83–1.23	0.47

*AL* Acute leukaemia, *ALL* Acute lymphoblastic leukaemia, *AML* Acute myeloid leukaemia, *N* Number of cases, *O* Observed number of cases, *E* Expected number of cases, *SIR* Standardized incidence ratio, 95% CI 95% Confidence interval

^a^ The crop density is defined as the ratio of the area used for the crop over the total area of the municipality (based on national agricultural census data). Separate models were used for each specific crop as well as for total crops

^b^ p-value of the tests (chi-square test of heterogeneity between SIRs in categories of crop density, test for departure from the log-linearity hypothesis and, test for the slope parameter in the linear Poisson regression model, H0: β ≤ 0 vs H1: β > 0)

^c^ SIRR (for Δ x = 10%) = Relative Standardized Incidence Ratio: multiplicative variation in the SIR for a 10% increase in the crop density derived from of a linear Poisson regression model, with adjustment on the size of urban unit (Paris *vs* other urban units) for AL and ALL

We examined the associations between ALL incidence rate and the potential ecological confounders by fitting a Poisson regression model with each variable separately (Additional Table 1). The size of the urban unit was associated with ALL incidence rate, with an 11% decrease in ALL incidence rate in the population of children living in the Paris urban unit at birth, compared to other urban units (SIRR = 0.89 95%CI [0.84–0.95]). ALL incidence rate also tended to be lower for children living in the most deprived municipalities, although statistical significance was not attained (SIRR = 0.96 95%CI [0.91–1.02]). As expected from our previous studies, residential UV radiation was positively associated with ALL (SIRR = 1.06 95%CI [1.01–1.12]).

These factors were also associated with crop densities (Additional Table 2). A large majority (83%) of children who lived in the Paris urban unit at birth was located in municipalities with less than 5% cropland, compared to 20% for the other urban units. For almost all crops, less than 0.5% of children in the Paris urban unit lived in municipalities in the highest crop density category. The categories with viticulture density ≥ 5% were more frequent in the municipalities with UV radiation > 105.5 J/cm^2^, with around 4% of the at-risk population in each category *vs* < 1% in municipalities with UV radiation ≤ 105.5 J/cm^2^. The same association was observed for arboriculture and sunflower densities. In contrast, UV radiation level tended to be negatively associated with rapeseed, dry vegetable and beet densities. The most deprived municipalities had a lower proportion of at-risk population in the first total crop density category. For specific crop densities, the distribution of the at-risk population in the last 4 categories was very similar in the most deprived municipalities and in other municipalities, except for straw cereals.

In sensitivity analyses on AL and ALL, we adjusted for residential UV radiation exposure and FDep 2006 index of the municipalities in addition to the size of urban unit already included in the regression models. The results did not differ from those of the main analyses (Table 4).

**Table 4:** Association between AL and ALL incidence rates and crop density^a^ in municipalities of residence at birth adjusted for size of urban unit, residential UV radiation exposure and French deprivation index (FDep), mainland France, RNCE, 1990–2015

	**AL (*N* = 8 747)**			**ALL (*N* = 7 236)**			**BCP-ALL (*N* = 6 970)**		
	SIRR	95% CI	p	SIRR	95% CI	p	SIRR	95% CI	p
**Total crops**									
10% increase in crop density	1.00	0.99–1.01	0.33	1.00	0.99–1.01	0.27	1.00	0.99–1.01	0.35
Size of UU (Paris UU vs. other UU)	0.89	0.84–0.95	< 0.01	0.91	0.84–0.97	0.005	0.90	0.83–0.96	< 0.01
UV (> 105.5 J/cm^2^ vs. ≤ 105.5 J/cm^2^)	1.01	0.96–1.06	0.79	1.03	0.98–1.09	0.29	1.04	0.98–1.10	0.20
Deprivation (Q5 vs. Q1-Q4)	0.95	0.90–1.00	0.06	0.95	0.90–1.01	0.11	0.94	0.89–1.00	0.06
**Viticulture**									
10% increase in crop density	1.01	0.98–1.05	0.2	1.01	0.98–1.05	0.23	1.01	0.98–1.05	0.25
Size of UU (Paris UU vs. other UU)	0.89	0.84–0.94	< 0.01	0.90	0.84–0.96	< 0.01	0.89	0.84–0.95	< 0.01
UV (> 105.5 J/cm^2^ vs. ≤ 105.5 J/cm^2^)	1.00	0.95–1.05	0.97	1.02	0.97–1.08	0.41	1.03	0.97–1.09	0.28
Deprivation (Q5 vs. Q1-Q4)	0.95	0.90–1.00	0.07	0.95	0.90–1.01	0.11	0.94	0.89–1.00	0.06
**Arboriculture**									
10% increase in crop density	0.87	0.78–0.98	0.99	0.88	0.78–0.99	0.98	0.87	0.76–0.98	0.98
Size of UU (Paris UU vs. other UU)	0.89	0.84–0.94	< 0.01	0.90	0.84–0.96	< 0.01	0.89	0.83–0.95	< 0.01
UV (> 105.5 J/cm^2^ vs. ≤ 105.5 J/cm^2^)	1.01	0.96–1.07	0.58	1.04	0.98–1.09	0.2	1.04	0.99–1.10	0.12
Deprivation (Q5 vs. Q1-Q4)	0.95	0.90–1.01	0.08	0.95	0.90–1.01	0.13	0.94	0.89–1.00	0.07
**Straw cereals**									
10% increase in crop density	1.01	0.99–1.03	0.14	1.01	0.99–1.03	0.11	1.01	0.99–1.04	0.11
Size of UU (Paris UU vs. other UU)	0.90	0.85–0.95	< 0.01	0.91	0.85–0.97	0.01	0.90	0.84–0.97	< 0.01
UV (> 105.5 J/cm^2^ vs. ≤ 105.5 J/cm^2^)	1.01	0.96–1.07	0.64	1.04	0.98–1.10	0.21	1.04	0.99–1.11	0.13
Deprivation (Q5 vs. Q1-Q4)	0.95	0.90–1.00	0.06	0.95	0.90–1.01	0.10	0.94	0.88–1.00	0.05
**Maize**									
10% increase in crop density	0.98	0.93–1.02	0.825	0.98	0.93–1.03	0.775	0.98	0.93–1.03	0.815
Size of UU (Paris UU vs. other UU)	0.88	0.83–0.94	< 0.01	0.89	0.84–0.95	< 0.01	0.88	0.83–0.95	< 0.01
UV (> 105.5 J/cm^2^ vs. ≤ 105.5 J/cm^2^)	1.00	0.95–1.05	0.95	1.02	0.97–1.08	0.39	1.03	0.98–1.09	0.28
Deprivation (Q5 vs. Q1-Q4)	0.95	0.90–1.00	0.07	0.95	0.90–1.01	0.11	0.94	0.89–1.00	0.06
**Rapeseed**									
10% increase in crop density	1.05	0.96–1.14	0.14	1.06	0.96–1.16	0.11	1.05	0.96–1.16	0.14
Size of UU (Paris UU vs. other UU)	0.89	0.84–0.95	< 0.01	0.90	0.85–0.97	< 0.01	0.90	0.84–0.96	< 0.01
UV (> 105.5 J/cm^2^ vs. ≤ 105.5 J/cm^2^)	1.01	0.96–1.06	0.71	1.03	0.98–1.09	0.24	1.04	0.98–1.10	0.16
Deprivation (Q5 vs. Q1-Q4)	0.95	0.90–1.00	0.07	0.95	0.90–1.01	0.12	0.94	0.89–1.00	0.06
**Sunflowers**									
10% increase in crop density	1.03	0.94–1.14	0.26	1.05	0.94–1.16	0.21	1.04	0.94–1.16	0.22
Size of UU (Paris UU vs. others UU)	0.89	0.84–0.94	< 0.01	0.90	0.84–0.96	< 0.01	0.89	0.84–0.95	< 0.01
UV (> 105.5 J/cm^2^ vs. ≤ 105.5 J/cm^2^)	1.00	0.96–1.05	0.89	1.03	0.97–1.08	0.37	1.03	0.98–1.09	0.25
Deprivation (Q5 vs. Q1-Q4)	0.95	0.90–1.00	0.06	0.95	0.90–1.01	0.11	0.94	0.89–1.00	0.06
**Potatoes**									
10% increase in crop density	1.11	0.97–1.26	0.06	1.11	0.96–1.28	0.08	1.12	0.97–1.29	0.06
Size of UU (Paris UU vs. other UU)	0.89	0.84–0.95	< 0.01	0.90	0.85–0.96	< 0.01	0.90	0.84–0.96	< 0.01
UV (> 105.5 J/cm^2^ vs. ≤ 105.5 J/cm^2^)	1.01	0.96–1.06	0.72	1.03	0.98–1.09	0.27	1.04	0.98–1.10	0.17
Deprivation (Q5 vs. Q1-Q4)	0.95	0.90–1.00	0.07	0.95	0.90–1.01	0.11	0.94	0.89–1.00	0.06
**Fresh vegetables**									
10% increase in crop density	1.07	0.96–1.19	0.12	1.04	0.92–1.18	0.255	1.03	0.91–1.17	0.31
Size of UU (Paris UU vs. other UU)	0.89	0.84–0.94	< 0.01	0.90	0.84–0.96	< 0.01	0.89	0.84–0.95	< 0.01
UV (> 105.5 J/cm^2^ vs. ≤ 105.5 J/cm^2^)	1.01	0.96–1.06	0.82	1.03	0.97–1.08	0.32	1.04	0.98–1.09	0.22
Deprivation (Q5 vs. Q1-Q4)	0.95	0.90–1.00	0.07	0.95	0.90–1.01	0.11	0.94	0.89–1.00	0.06
**Dry vegetables**									
10% increase in crop density	1.02	0.89–1.18	0.37	1.00	0.86–1.17	0.50	1.00	0.85–1.17	0.51
Size of UU (Paris UU vs. other UU)	0.89	0.84–0.94	< 0.01	0.90	0.84–0.96	< 0.01	0.89	0.83–0.95	< 0.01
UV (> 105.5 J/cm^2^ vs. ≤ 105.5 J/cm^2^)	1.01	0.96–1.06	0.81	1.03	0.97–1.08	0.33	1.03	0.98–1.09	0.22
Deprivation (Q5 vs. Q1-Q4)	0.95	0.90–1.00	0.07	0.95	0.90–1.01	0.11	0.94	0.89–1.00	0.06
**Beet**									
10% increase in crop density	0.97	0.88–1.06	0.76	0.97	0.88–1.07	0.73	0.97	0.88–1.08	0.70
Size of UU (Paris UU vs. other UU)	0.89	0.84–0.94	< 0.01	0.90	0.84–0.95	< 0.01	0.89	0.83–0.95	< 0.01
UV (> 105.5 J/cm^2^ vs. ≤ 105.5 J/cm^2^)	1.00	0.95–1.05	0.93	1.02	0.97–1.08	0.38	1.03	0.98–1.09	0.26
Deprivation (Q5 vs. Q1-Q4)	0.95	0.90–1.00	0.07	0.95	0.90–1.01	0.12	0.94	0.89–1.00	0.06

*AL* Acute leukaemia, *ALL* Acute lymphoblastic leukaemia, *BCP-ALL* B-cell precursors ALL, *N* Number of cases

^a^ The total crop density and the specific crop density in a municipality are defined as the ratio of the total area used for agriculture and the area used for the specific crop, respectively, over the total area of the municipality (based on national agricultural census data). Separate models were used for each specific crop as well as for total crops

p: p-value associated with the regression coefficients in the multivariate Poisson regression model

Analyses performed by excluding the Paris urban unit, or municipalities from the most populated urban units (population > 100,000) did not change the results (Additional Table 3).

When we stratified the analyses by two age groups (0–6 and 7–14 years old), we found a significant positive log-linear association between rapeseed density and ALL incidence among 7–14-year olds (SIRR = 1.29 95%CI [1.12–1.49] per 10% increase in rapeseed density, Additional Table 4). The numbers of cases in the municipalities with the highest rapeseed densities were, however, quite limited (25–30 expected cases) and the SIRs were heterogeneous, with values greater than one in the third and last categories (Additional Fig. 2). For the other crops, the results remained stable by age-group (Additional Table 4). The analyses with viticulture restricted to children born during the period 1990–2004 resulted in the same conclusion (data not shown).

## Discussion

We investigated the association between total and specific-crop densities in the municipalities of residence at birth and childhood leukaemia incidence rates in the population of children born in 1990–2015. In all, we did not observe any statistically significant positive association between total childhood AL, ALL and AML incidence rates and any crop type. Interestingly, the incidence rate of AL was slightly higher in children living at birth in the municipalities with the highest viticulture densities (SIR = 1.25 95%CI [1.01–1.54] in the highest semi-quartile of exposure), which was in line with our study on residences at diagnosis [12].

Based on the same methodology and same agricultural data, we evidenced a log-linear increase in ALL incidence rate over the period 1990–2014 with increasing viticulture density in the municipality of residence at diagnosis (SIRR = 1.04 95%CI [1.00–1.06] per 10% increase) and an increase in ALL incidence rate in the municipalities with the highest viticulture density (i.e. with more than a quarter of their area used for viticulture, SIR = 1.17 95%CI [1.01–1.35]) [12]. Although the result of the present study is not statistically significant, and less marked than in our previous study, it provides some support for the hypothesis that there is a positive association between childhood AL incidence and viticulture density in the municipalities. However, the weakness of the association does not suggest that our previous results can be explained by prenatal exposures.

Viticulture is all the more of interest in that vines are a permanent crop subject to many pesticide treatments, particularly fungicidal treatments. A few studies have investigated the role of residential exposure to agricultural pesticides in the occurrence of childhood AL using proximity to croplands as a surrogate (Additional Table 5). Most of the studies were conducted in the USA. With a large number of cases (6,168 AL), Carozza et al. [11] considered the percentage cropland devoted to farming at the county level and showed a positive association between total cropland (≥ 60% of county total acreage *vs* < 20%) and ALL and AML risk (OR = 1.3 95%CI [1.1–1.4], and OR = 1.8 95% CI [1.4–2.3], respectively). The authors also reported positive associations with some specific crops (maize and soya bean densities for AML, oats density for ALL). In a study by Booth et al. [10] conducted in 6 Midwestern states on the county scale, 0–4-year AL and ALL incidence rates were associated with dry beans and sugar beet density in exposure–response analyses. A positive association between 0–4-year AML incidence rates and oats density was also reported. More recently, a study conducted in California reported a greater ALL risk in children residing close to plant nurseries at birth (OR for < 75 m *vs* ≥ 600 m of 3.09 95% CI [1.14–8.34] [7]). Conversely, three Texan studies (9,13,24) did not evidence any association between childhood AL and agricultural area in the county of residence at birth [13, 24] or within a 1000-m buffer around the geocoded address of residence at birth [9]. In addition to our previous ecological study [12], three studies have been conducted in Europe [6, 8, 19]. In Italy, in a case–control study with 111 AL cases and 444 matched controls, Malagoli et al. did not find any association between AL risk and arable crop, orchard, vineyard or vegetable densities within 100 m from the geocoded addresses of residence [6], while, in Spain, Gomez-Barroso et al. reported an elevated risk of leukaemia (1,062 cases) with total crop density and several specific crop densities (arable land or permanently irrigated land; rice fields; heterogeneous agricultural areas) within 1-km buffers [8]. The study also found strong associations with other childhood cancer types; it is unclear whether those results may be due to different addresses being used for the cases (at diagnosis) and controls (at birth). A recent Danish cohort study [19] reported a twofold increase in leukaemia risk (61 AL cases) for children whose mothers lived close to agricultural areas during pregnancy (≥ 24 ha of total agricultural land within a 500-m buffer), in particular when grass/clover, peas and maize crops were present.

Several studies thus reported positive associations between childhood AL risk and proximity to cropland, particularly around diagnosis [6, 8, 10–12, 19]. However, the estimates of the reported associations were sometimes imprecise due to the limited numbers of cases [6, 19]. In addition, several studies reported positive associations for total crop density [8, 11, 19], which may not be a relevant surrogate for pesticide exposure because of the heterogeneity of the crops included, especially in terms of pesticide use. It is difficult to compare our results with those from other countries because of differences in agricultural areas and practices [25]. For example, in the USA, positive associations were reported with dry beans, sugar beet [10], oats [10, 11], maize and soya beans [11], which are less common crops in France [25].

We used agricultural census data to estimate crop densities in the municipalities of residence at birth, as indicators of potential exposure to agricultural pesticides. A limitation consists in the fact that the census locates the crops in the municipality of the farm headquarters, while the crops may be located in neighbouring municipalities. However, the resulting misclassifications most likely affect similar agricultural areas close to each other and preserve contrasts between exposed and unexposed municipalities. Another limitation is that we assigned the agricultural census closest to the year of birth, which may have induced misclassification for rotational crops when the years of census and birth differed. This limitation should not affect the classification of permanent crops like vineyards and arboriculture. Moreover, the results remained unchanged in the sensitivity analyses restricted to children born in 1990–2004, before many vines were uprooted.

The agricultural censuses do not distinguish organically-farmed fields from conventionally-farmed fields. However, organically-farmed fields constitute only a small fraction of the agricultural area of France; they accounted for 9.5% of the total agricultural area in 2019 [26] and probably less during our study period since organic farming was less widespread. Moreover, two surveys of agricultural practices showed that more than 90% of the areas covered by the crops we considered in our study had received at least one pesticide treatment in 2006 and 2011 [27, 28].

Adjustments for the degree of urbanization, deprivation and UV radiation, which were associated with childhood ALL incidence rate at the municipality level in our previous studies, did not change the result. We were unable to take individual factors like domestic pesticide use and parental agricultural occupation into account. However, the proportion of children with a parent occupationally exposed to pesticides was estimated to be very low in a French national case–control study [5]. Maternal use of pesticides during pregnancy is much more common, about 40% for control mothers in two French case–control studies [29] but not restricted to agricultural areas. Residential exposure to traffic-related air pollution, which was associated with the risk of childhood AML in previous French studies [30, 31], could not be accounted for. However, the results were unchanged after exclusion of the municipalities in urban units with a population greater than 100,000, where those associations were observed.

Our study has several strengths. A major asset is that our findings are based on a large number of cases, identified from a population-based registry. The high degree of completeness, avoided selection biases, and the high standard of diagnosis classifications and high reliability of addresses (3% missing addresses) minimised misclassifications. Another asset is the use of agricultural census data, collected on an exhaustive basis and providing the detailed distribution of ten types of crops on the fine scale of the municipality for the entire country. The crop types are known to be quite different in terms of average annual number of pesticide treatments, percentage of area treated, and main target pesticide, with variations between time periods and regions [27, 28]. A few Californian studies benefitted from the Pesticides Use Reporting (PUR) system, with a large database on pesticides applied to crops in the state of California, to investigate for associations with childhood leukaemia [14–18]. Some positive associations with specific substances or classes of pesticide have been reported, with, however, heterogeneous results: AL risk was thus associated with high use of propargite near the residence at diagnosis in Reynold et al. 2002 [16]; a moderate lifetime averaged use of insecticides and fumigants in the vicinity of the addresses of residence in Rull et al. [17]; the use of metam sodium and dicofol near the residence at birth for children aged 0–4 years in Reynold et al. 2005 [15]; and the uses of any carcinogenic pesticide, several chemical classes or individual pesticides near the residence at birth for children aged less than 6 years in Park et al. [14].

Our next step will be to conduct a large case–control study using geocoded addresses and a geographical information system in order to evaluate, as precisely as possible, the presence of cropland in the vicinity of the residential addresses, with a particular focus on viticulture. Elucidating the relationship between the ecological and individual crop proximity indicators will be of great importance. Future challenges will then consist in enhanced assessment of the role of agricultural pesticide exposure and identification of the specific substances potentially involved in childhood leukaemia.

## Conclusion

The present study did not evidence statistically significant associations between the density of crops in the municipality of birth and the incidence rates of childhood AL. The slightly higher ALL incidence rate in children born in the municipalities with the highest viticulture densities may reflect the association that we previously observed at diagnosis. But, overall, our results do not support the hypothesis that prenatal exposure to neighbouring agricultural activity, particularly viticulture, plays a role in childhood leukaemia.

## Supplementary Information

**Additional file 1:** **Additional Figure 1.** Annual standardized incidence ratio (SIR) of childhood AL, RNCE, 1990-2015, mainland France.

**Additional file 2:** **Additional Figure 2.** Standardized incidence ratio (SIR) of acute lymphoblastic leukaemia (ALL) in the municipalities of residence at birth grouped into rapeseed density^a^ categories, for children aged 7-14 years (mainland France, RNCE, 1990–2015).

**Additional file 3:** **Additional Table 1.** Association between the incidence rate of childhood acute lymphoblastic leukaemia and three potential ecological confounders characterising the municipality of residence at birth: size of urban unit, residential UV radiation exposure, and a socioeconomic deprivation index (RNCE, 1990–2015).

**Additional file 4:** **Additional Table 2.** Distribution of the at-risk population ^a^ over 1990-2015 by category of crop density and potential confounding factor stratum (size of urban unit, residential UV radiation exposure, and a French deprivation index), children born in 1990-2015, mainland France.

**Additional file 5:** **Additional Table 3.** Association between AL incidence rate and crop density^a^ in municipalities of residence at birth with restriction to municipalities situated outside of the Paris urban unit, and municipalities in urban units with a population of less than 100,000, mainland France, RNCE, 1990–2015.

**Additional file 6:** **Additional Table 4.** Association between ALL and BCP-ALL incidence rates and crop densities^a^ in the municipalities of residence at birth, stratified by age group (mainland France, RNCE, 1990–2015).

**Additional file 7:** **Additional Table 5.** Bibliography.

## Data Availability

Data and programs supporting the results of the study have been archived by the Inserm EPICEA team, in compliance with the general data protection regulation.

## Abbreviations

AL: Acute leukaemia
ALL: Acute lymphoblastic leukaemia
AML: Acute myeloid leukaemia
BCP-ALL: B-cell precursors ALL
FDep: French deprivation index
INSEE: French national institute for statistics and economic studies
RNCE: French national registry of childhood cancer
SIR: Standardized Incidence Ratio
SIRR: Relative Standardized Incidence Ratio
