# Supplementary material for: Residential proximity to croplands at birth and childhood leukaemia
Source: Environ Health. 2022 Oct 27;21:103. doi: 10.1186/s12940-022-00909-0 (PMC9615229; doi:10.1186/s12940-022-00909-0)
Supplement: Supplementary file 1 — Additional file 1: Additional Figure 1. Annual standardized incidence ratio (SIR) of childhood AL, RNCE, 1990-2015, mainland France. [file 12940_2022_909_MOESM1_ESM.docx]

Additional Figure 1: Annual standardized incidence ratio (SIR) of childhood AL, RNCE, 1990-2015, mainland France


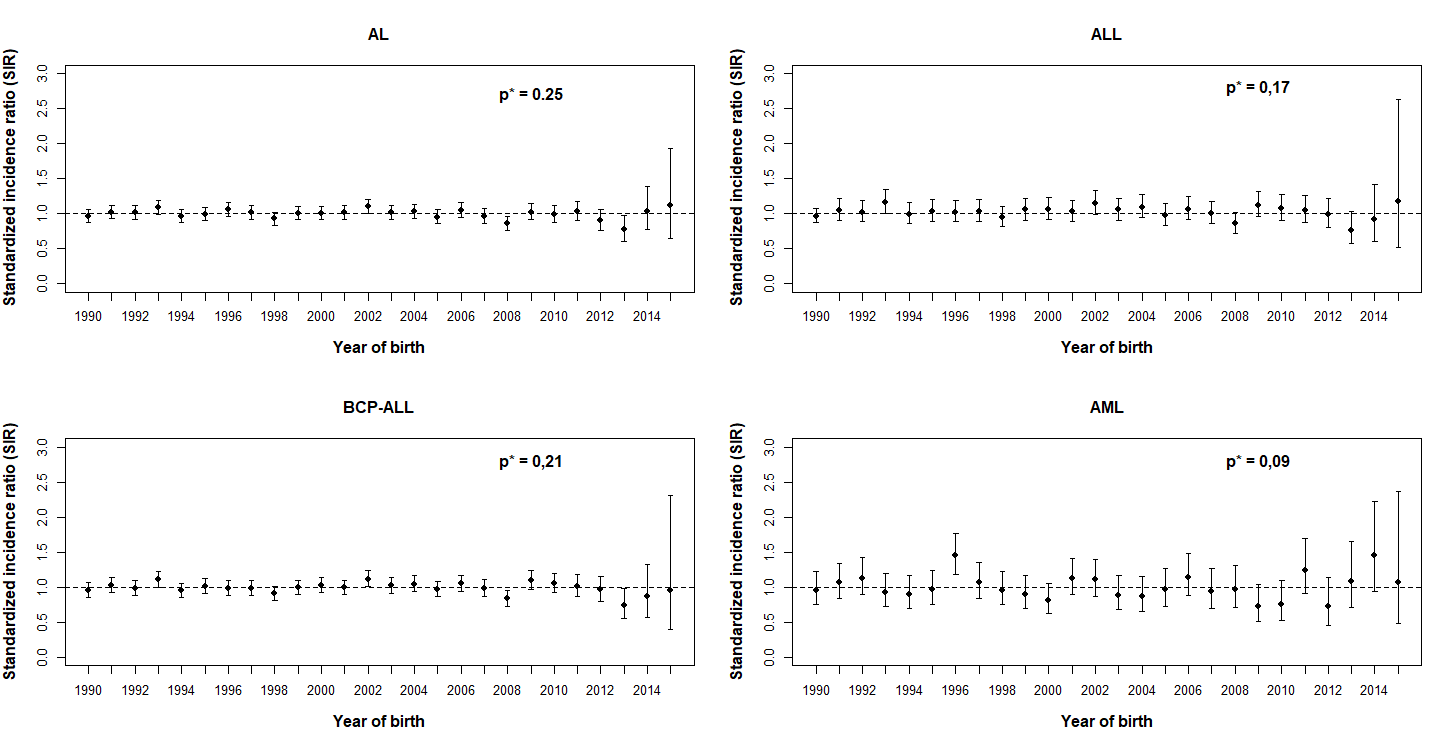


AL: Acute leukaemia; ALL: Acute lymphoblastic leukaemia; BCP-ALL: B-cell precursor ALL; AML: Acute myeloid leukaemia; SIR= Standardized incidence ratio

*p-value of the chi-square test of heterogeneity of years of birthof heterogeneity of SIR of rapeseed density categories
