# Supplementary material for: Residential proximity to croplands at birth and childhood leukaemia
Source: Environ Health. 2022 Oct 27;21:103. doi: 10.1186/s12940-022-00909-0 (PMC9615229; doi:10.1186/s12940-022-00909-0)
Supplement: Supplementary file 2 — Additional file 2: Additional Figure 2. Standardized incidence ratio (SIR) of acute lymphoblastic leukaemia (ALL) in the municipalities of residence at birth grouped into rapeseed densitya categories, for children aged 7-14 years (mainland France, RNCE, 1990–2015). [file 12940_2022_909_MOESM2_ESM.docx]

Additional Figure 2: Standardized incidence ratio (SIR) of acute lymphoblastic leukaemia (ALL) in the municipalities of residence at birth grouped into rapeseed density^a^ categories, for children aged 7-14 years (mainland France, RNCE, 1990–2015)


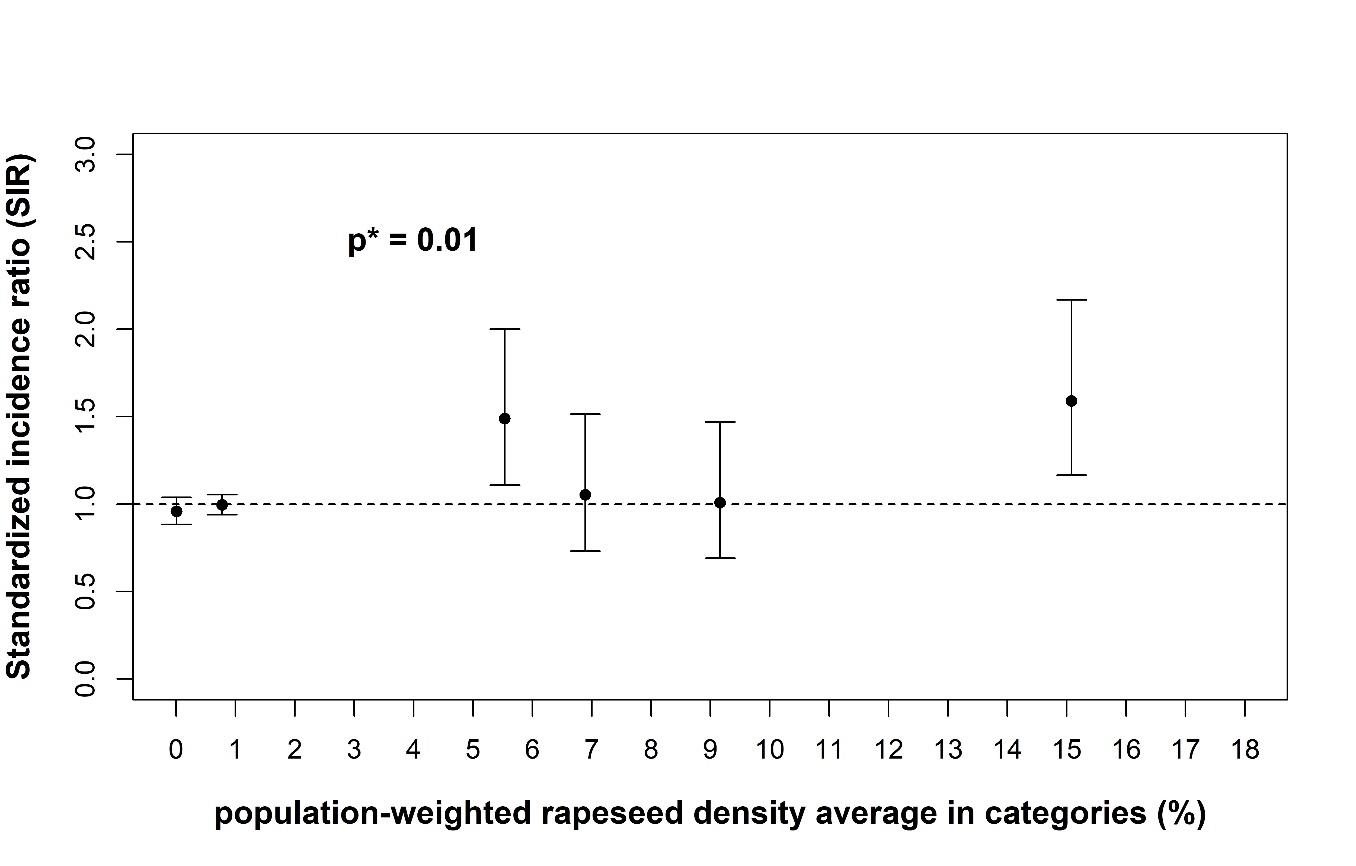


^a^ The rapeseed density in a municipality is defined as the ratio of the total area used for rapeseed over the total area of the municipality

*p-value of the chi-square test of heterogeneity of SIR of rapeseed density categories
