# Supplementary material for: Residential proximity to croplands at birth and childhood leukaemia
Source: Environ Health. 2022 Oct 27;21:103. doi: 10.1186/s12940-022-00909-0 (PMC9615229; doi:10.1186/s12940-022-00909-0)
Supplement: Supplementary file 3 — Additional file 3: Additional Table 1. Association between the incidence rate of childhood acute lymphoblastic leukaemia and three potential ecological confounders characterising the municipality of residence at birth: size of urban unit, residential UV radiation exposure, and a socioeconomic deprivation index (RNCE, 1990–2015). [file 12940_2022_909_MOESM3_ESM.docx]

Additional Table 1: Association between the incidence rate of childhood acute lymphoblastic leukaemia and three potential ecological confounders characterising the municipality of residence at birth: size of urban unit, residential UV radiation exposure, and a socioeconomic deprivation index (RNCE, 1990–2015).

|  | O | E | SIRR | 95% CI | p^a^ |
| --- | --- | --- | --- | --- | --- |
| Size of urban unit |  |  |  |  |  |
| All urban units except Paris | 5,877 | 5,750.0 | 1 | Ref |  |
| Paris urban unit | 1,359 | 1,486.0 | 0.89 | 0.84-0.95 | <0.001 |
| UV radiation |  |  |  |  |  |
| ≤105.5 J/cm^2^ | 5,059 | 5,150.5 | 1 | Ref |  |
| >105.5 J/cm^2^ | 2,177 | 2,085.5 | 1.06 | 1.01-1.12 | 0.02 |
| FDep 2006 |  |  |  |  |  |
| Least deprived municipalities | 5,828 | 5,786.0 | 1 | Ref |  |
| Most deprived municipalities | 1,408 | 1,450.0 | 0.96 | 0.91-1.02 | 0.20 |

ALL: Acute lymphoblastic leukaemia; O: Observed number of cases; E: Expected number of cases; SIRR: Relative Standardized Incidence Ratio; 95% CI: 95% Confidence Interval

^a^p-value of the chi-square test of heterogeneity between categories of size of urban unit, categories of UV radiation, categories of FDep 2006 included in a one regression model for each variable separately
