# Supplementary material for: Residential proximity to croplands at birth and childhood leukaemia
Source: Environ Health. 2022 Oct 27;21:103. doi: 10.1186/s12940-022-00909-0 (PMC9615229; doi:10.1186/s12940-022-00909-0)
Supplement: Supplementary file 4 — Additional file 4: Additional Table 2. Distribution of the at-risk population a over 1990-2015 by category of crop density and potential confounding factor stratum (size of urban unit, residential UV radiation exposure, and a French deprivation index), children born in 1990-2015, mainland France. [file 12940_2022_909_MOESM4_ESM.docx]

Additional Table 2: Distribution of the at-risk population ^a^ over 1990-2015 by category of crop density and potential confounding factor stratum (size of urban unit, residential UV radiation exposure, and a French deprivation index), children born in 1990-2015, mainland France.

|  | All urban units except Paris | | Municipalities  In Paris urban unit | |  | Municipalities  with UV radiation  ≤105.5 J/cm^2^ | | Municipalities  with UV radiation  >105.5 J/cm^2^ | |  | Other  municipalities  (Q1-Q4 FDep) | | Most deprived  Municipalities (Q5 Fdep) | |
| --- | --- | --- | --- | --- | --- | --- | --- | --- | --- | --- | --- | --- | --- | --- |
| Total crops | population ^a^ | % | population ^a^ | % |  | population ^a^ | % | population ^a^ | % |  | population ^a^ | % | population ^a^ | % |
| Total crops <5% | 1,275,212 | 19.9 | 1,368,131 | 82.9 |  | 2,063,502 | 36.0 | 579,840 | 25.1 |  | 2,297,836 | 35.7 | 345,506 | 21.4 |
| [5.0-18.7[ | 1,215,502.6 | 19.0 | 132,252 | 8.0 |  | 833,580 | 14.5 | 514,174 | 22.2 |  | 1,050,127 | 16.3 | 297,627 | 18.4 |
| [18.7-37.4[ | 1,259,605.3 | 19.7 | 93,227 | 5.6 |  | 875,026 | 15.3 | 477,806 | 20.6 |  | 1,068,226 | 16.6 | 284,606 | 17.6 |
| [37.4-61.0[ | 1,303,401.3 | 20.4 | 46,897 | 2.8 |  | 895,686 | 15.6 | 454,612 | 19.6 |  | 1,027,139 | 16.0 | 323,160 | 20.0 |
| ≥61% | 1,340,279.2 | 21.0 | 9,977 | 0.6 |  | 1,062,615 | 18.5 | 287,640 | 12.4 |  | 986,421 | 15.3 | 363,835 | 22.5 |
| Viticulture |  |  |  |  |  |  |  |  |  |  |  |  |  |  |
| Total crops <5% | 1,275,212.0 | 19.9 | 1,368,131 | 82.9 |  | 2,063,502 | 36.0 | 579,840 | 25.1 |  | 2,297,836 | 35.7 | 345,506 | 21.4 |
| Total crops ≥5% and  viticulture <5% | 4,607,734.6 | 72.1 | 282,355 | 17.1 |  | 3,537,544 | 61.7 | 1,352,545 | 58.4 |  | 3,712,300 | 57.7 | 1,177,789 | 72.9 |
| [5.0-10.0[ | 127,698.5 | 2.0 | 0 | 0.0 |  | 36,080 | 0.6 | 91,617 | 4.0 |  | 102,180 | 1.6 | 25,517 | 1.6 |
| [10.0-16.2[ | 127,795.9 | 2.0 | 0 | 0.0 |  | 30,058 | 0.5 | 97,737 | 4.2 |  | 105,345 | 1.6 | 22,450 | 1.4 |
| [16.2-24.7[ | 129,213.6 | 2.0 | 0 | 0.0 |  | 38,161 | 0.7 | 91,052 | 3.9 |  | 107,480 | 1.7 | 21,732 | 1.3 |
| ≥24.7 | 126,345.8 | 2.0 | 0 | 0.0 |  | 25,064 | 0.4 | 101,281 | 4.4 |  | 104,608 | 1.6 | 21,737 | 1.3 |
| Arboriculture |  |  |  |  |  |  |  |  |  |  |  |  |  |  |
| Total crops <5% | 1,275,212.0 | 19.9 | 1,368,131 | 82.9 |  | 2,063,502 | 36.0 | 579,840 | 25.1 |  | 2,297,836 | 35.7 | 345,506 | 21.4 |
| Total crops ≥5% and  arboriculture <5% | 4,934,655.4 | 77.2 | 267,711 | 16.2 |  | 3,619,358 | 63.2 | 1,583,008 | 68.4 |  | 3,974,033 | 61.8 | 1,228,333 | 76.1 |
| [5.0-6.4[ | 45,878.2 | 0.7 | 4,579 | 0.3 |  | 14,252 | 0.2 | 36,205 | 1.6 |  | 43,367 | 0.7 | 7,090 | 0.4 |
| [6.4-9.34[ | 41,651.3 | 0.7 | 6,705 | 0.4 |  | 16,466 | 0.3 | 31,890 | 1.4 |  | 38,251 | 0.6 | 10,105 | 0.6 |
| [9.4-13.1[ | 46,146.8 | 0.7 | 796 | 0.0 |  | 10,459 | 0.2 | 36,484 | 1.6 |  | 38,705 | 0.6 | 8,237 | 0.5 |
| ≥13.1 | 50,456.7 | 0.8 | 2,562 | 0.2 |  | 6,372 | 0.1 | 46,646 | 2.0 |  | 37,557 | 0.6 | 15,461 | 1.0 |
| Straw cereals |  |  |  |  |  |  |  |  |  |  |  |  |  |  |
| Total crops <5% | 1,275,212.0 | 19.9 | 1,368,131 | 82.9 |  | 2,063,502 | 36.0 | 579,840 | 25.1 |  | 2,297,836 | 35.7 | 345,506 | 21.4 |
| Total crops ≥5% and  straw cereals <5% | 1,982,259 | 31.0 | 94,532 | 5.7 |  | 1,098,982 | 19.2 | 977,809 | 42.3 |  | 1,662,548 | 25.9 | 414,242 | 25.7 |
| [5.0-8.8[ | 780,930 | 12.2 | 50,182 | 3.0 |  | 536,565 | 9.4 | 294547 | 12.7 |  | 635,606 | 9.9 | 195506 | 12.1 |
| [8.8-14.2[ | 778,900 | 12.2 | 49,922 | 3.0 |  | 616,994 | 10.8 | 211829 | 9.2 |  | 631,110 | 9.8 | 197713 | 12.2 |
| [14.2-23.8[ | 779,326 | 12.2 | 53,337 | 3.2 |  | 667,325 | 11.6 | 165338 | 7.1 |  | 604,631 | 9.4 | 228,032 | 14.1 |
| ≥23.8 | 797,370 | 12.5 | 34,381 | 2.1 |  | 747,042 | 13.0 | 84709 | 3.7 |  | 598,018 | 9.3 | 233,733 | 14.5 |
| Maize |  |  |  |  |  |  |  |  |  |  |  |  |  |  |
| Total crops <5% | 1,275,212 | 19.9 | 1,368,131 | 82.9 |  | 2,063,502 | 36.0 | 579840 | 25.1 |  | 2,297,836 | 35.7 | 345,506 | 21.4 |
| Total crops ≥5% and  maize <5% | 3,950,154 | 61.8 | 273,762 | 16.6 |  | 2,705,669 | 47.2 | 1518247 | 65.6 |  | 3,263,423 | 50.8 | 960,493 | 59.5 |
| [5.0-7.0[ | 289,746 | 4.5 | 5,210 | 0.3 |  | 229,476 | 4.0 | 65481 | 2.8 |  | 209,876 | 3.3 | 85,080 | 5.3 |
| [7.03-9.8[ | 292,675 | 4.6 | 853 | 0.1 |  | 243,480 | 4.2 | 50048 | 2.2 |  | 219,705 | 3.4 | 73,823 | 4.6 |
| [9.83-14.4[ | 292,359 | 4.6 | 2,060 | 0.1 |  | 247,427 | 4.3 | 46993 | 2.0 |  | 213,015 | 3.3 | 81,405 | 5.0 |
| ≥14.4 | 293,852 | 4.6 | 467 | 0.0 |  | 240,856 | 4.2 | 53463 | 2.3 |  | 225,895 | 3.5 | 68,424 | 4.2 |
| Rapeseed |  |  |  |  |  |  |  |  |  |  |  |  |  |  |
| Total crops <5% | 1,275,212 | 19.9 | 1,368,131 | 82.9 |  | 2,063,502 | 36.0 | 579840 | 25.1 |  | 2,297,836 | 35.7 | 345,506 | 21.4 |
| Total crops ≥5% and  rapeseed<5% | 4,649,352 | 72.7 | 247,364 | 15.0 |  | 3,196,054 | 55.8 | 1700662 | 73.5 |  | 3,737,106 | 58.1 | 1,159,610 | 71.8 |
| [5.0-6.1[ | 114,609 | 1.8 | 12,593 | 0.8 |  | 114,156 | 2.0 | 13046 | 0.6 |  | 101,559 | 1.6 | 25,644 | 1.6 |
| [6.1-7.9[ | 116,390 | 1.8 | 8,844 | 0.5 |  | 116,660 | 2.0 | 8574 | 0.4 |  | 100,657 | 1.6 | 24,577 | 1.5 |
| [7.9-11.0[ | 115,585 | 1.8 | 9,270 | 0.6 |  | 115,455 | 2.0 | 9399 | 0.4 |  | 98,012 | 1.5 | 26,843 | 1.7 |
| ≥11.0 | 122,850 | 1.9 | 4,282 | 0.3 |  | 124,582 | 2.2 | 2550 | 0.1 |  | 94,578 | 1.5 | 32,553 | 2.0 |
| Sunflowers |  |  |  |  |  |  |  |  |  |  |  |  |  |  |
| Total crops <5% | 1,275,212 | 19.9 | 1,368,131 | 82.9 |  | 2,063,502 | 36.0 | 579840 | 25.1 |  | 2,297,836 | 35.7 | 345,506 | 21.4 |
| Total crops ≥5% and  sunflowers <5% | 4,793,265 | 75.0 | 282,173 | 17.1 |  | 3,523,540 | 61.5 | 1,551,897 | 67.1 |  | 3,871,211 | 60.2 | 1,204,226 | 74.6 |
| [5.0-6.2[ | 81,148 | 1.3 | 137 | 0.0 |  | 39,918 | 0.7 | 41,366 | 1.8 |  | 69,545 | 1.1 | 11,739 | 0.7 |
| [6.2-8.2[ | 80,681 | 1.3 | 28 | 0.0 |  | 36,796 | 0.6 | 43,914 | 1.9 |  | 63,109 | 1.0 | 17,600 | 1.1 |
| [8.2-11.9[ | 82,275 | 1.3 | 0 | 0.0 |  | 40,152 | 0.7 | 42,123 | 1.8 |  | 64,151 | 1.0 | 18,123 | 1.1 |
| ≥11.9 | 81,418 | 1.3 | 16 | 0.0 |  | 26,501 | 0.5 | 54,932 | 2.4 |  | 63,896 | 1.0 | 17,537 | 1.1 |
| Potatoes |  |  |  |  |  |  |  |  |  |  |  |  |  |  |
| Total crops <5% | 1,275,212 | 19.9 | 1,368,131 | 82.9 |  | 2,063,502 | 36.0 | 579,840.5 | 25.1 |  | 2,297,836 | 35.7 | 345,506 | 21.4 |
| Total crops ≥5% and  potatoes <5% | 4,970,211 | 77.7 | 281,909 | 17.1 |  | 3,521,756 | 61.5 | 1,730,365 | 74.8 |  | 4,026,127 | 62.6 | 1,225,994 | 75.9 |
| [5.0-6.3[ | 36,605 | 0.6 | 416 | 0.0 |  | 35,656 | 0.6 | 1,365 | 0.1 |  | 24,724 | 0.4 | 12,297 | 0.8 |
| [6.3-8.5[ | 37,490 | 0.6 | 28 | 0.0 |  | 36,454 | 0.6 | 1,064 | 0.0 |  | 26,606 | 0.4 | 10,912 | 0.7 |
| [8.47-12.6[ | 36,924 | 0.6 | 0 | 0.0 |  | 36,245 | 0.6 | 679 | 0.0 |  | 26,270 | 0.4 | 10,654 | 0.7 |
| ≥12.6 | 37,557 | 0.6 | 0 | 0.0 |  | 36,796 | 0.6 | 760 | 0.0 |  | 28,187 | 0.4 | 9,370 | 0.6 |
| Fresh vegetables |  |  |  |  |  |  |  |  |  |  |  |  |  |  |
| Total crops <5% | 1,275,212 | 19.9 | 1,368,131 | 82.9 |  | 2,063,502 | 36.0 | 579,840 | 25.1 |  | 2,297,836 | 35.7 | 345,506 | 21.4 |
| Total crops ≥5% and  fresh vegetables <5% | 4,925,576 | 77.0 | 264,443 | 16.0 |  | 3,521,891 | 61.5 | 1,668,128 | 72.1 |  | 3,974,373 | 61.8 | 1,215,646 | 75.3 |
| [5.0-6.11[ | 43,734 | 0.7 | 8,697 | 0.5 |  | 36,374 | 0.6 | 16,057 | 0.7 |  | 37,837 | 0.6 | 14,594 | 0.9 |
| [6.11-7.7[ | 52,033 | 0.8 | 980 | 0.1 |  | 30,993 | 0.5 | 22,020 | 1.0 |  | 34,137 | 0.5 | 18,876 | 1.2 |
| [7.7-11.5[ | 49,015 | 0.8 | 2,954 | 0.2 |  | 37,223 | 0.6 | 14,747 | 0.6 |  | 42,869 | 0.7 | 9,101 | 0.6 |
| ≥11.5 | 48,427 | 0.8 | 5,279 | 0.3 |  | 40,427 | 0.7 | 13,279 | 0.6 |  | 42,697 | 0.7 | 11,009 | 0.7 |
| Dry vegetables |  |  |  |  |  |  |  |  |  |  |  |  |  |  |
| Total crops <5% | 1,275,212 | 19.9 | 1,368,131 | 82.9 |  | 2,063,502 | 36.0 | 579,840 | 25.1 |  | 2,297,836 | 35.7 | 345,506 | 21.4 |
| Total crops ≥5% and  dry vegetables <5% | 4,876,784 | 76.3 | 275,428 | 16.7 |  | 3,434,341 | 59.9 | 1,717,871 | 74.2 |  | 3,945,408 | 61.4 | 1,206,804 | 74.7 |
| [5.0-5.9[ | 59,687 | 0.9 | 2,249 | 0.1 |  | 57,049 | 1.0 | 4,887 | 0.2 |  | 42,494 | 0.7 | 19,442 | 1.2 |
| [5.9-7.2[ | 59,874 | 0.9 | 2,581 | 0.2 |  | 58,278 | 1.0 | 4,177 | 0.2 |  | 46,972 | 0.7 | 15,483 | 1.0 |
| [7.23-9.8[ | 60,265 | 0.9 | 1,946 | 0.1 |  | 58,010 | 1.0 | 4,201 | 0.2 |  | 47,414 | 0.7 | 14,797 | 0.9 |
| ≥9.8 | 62,176 | 1.0 | 149 | 0.0 |  | 59,229 | 1.0 | 3,096 | 0.1 |  | 49,624 | 0.8 | 12,700 | 0.8 |
| Beet |  |  |  |  |  |  |  |  |  |  |  |  |  |  |
| Total crops <5% | 1,275,212 | 19.9 | 1,368,131 | 82.9 |  | 2,063,502 | 36.0 | 579,840 | 25.1 |  | 2,297,836 | 35.7 | 345,506 | 21.4 |
| Total crops ≥5% and  beet <5% | 4,764,095 | 74.5 | 261,450 | 15.8 |  | 3,293,044 | 57.5 | 1,732,501 | 74.9 |  | 3,895,240 | 60.6 | 1,130,305 | 70.0 |
| [5.0-6.9[ | 89,722 | 1.4 | 4,281 | 0.3 |  | 93,335 | 1.6 | 668 | 0.0 |  | 61,926 | 1.0 | 32,077 | 2.0 |
| [6.9-9.0[ | 87,325 | 1.4 | 6,832 | 0.4 |  | 93,564 | 1.6 | 594 | 0.0 |  | 58,922 | 0.9 | 35,236 | 2.2 |
| [9.04-13.0[ | 90,996 | 1.4 | 2,533 | 0.2 |  | 93,165 | 1.6 | 364 | 0.0 |  | 56,384 | 0.9 | 37,145 | 2.3 |
| ≥13.0 | 86,648 | 1.4 | 7,257 | 0.4 |  | 93,800 | 1.6 | 105 | 0.0 |  | 59,441 | 0.9 | 34,464 | 2.1 |

^a^ Annual average at-risk population (person-year)
