# Supplementary material for: Residential proximity to croplands at birth and childhood leukaemia
Source: Environ Health. 2022 Oct 27;21:103. doi: 10.1186/s12940-022-00909-0 (PMC9615229; doi:10.1186/s12940-022-00909-0)
Supplement: Supplementary file 5 — Additional file 5: Additional Table 3. Association between AL incidence rate and crop densitya in municipalities of residence at birth with restriction to municipalities situated outside of the Paris urban unit, and municipalities in urban units with a population of less than 100,000, mainland France, RNCE, 1990–2015. [file 12940_2022_909_MOESM5_ESM.docx]

Additional Table 3: Association between AL incidence rate and crop density^a^ in municipalities of residence at birth with restriction to municipalities situated outside of the Paris urban unit, and municipalities in urban units with a population of less than 100,000, mainland France, RNCE, 1990–2015

|  |  | Municipalities out of Paris urban unit | | | | | Municipalities with population less than 100 000 | | | | |
| --- | --- | --- | --- | --- | --- | --- | --- | --- | --- | --- | --- |
|  | **Q3** | **E(Q3)** | **SIRR** | **95%CI** | **p_loglin_** | **p_slope_** | **E(Q3)** | **SIRR** | **95%CI** | **p_loglin_** | **p_slope_** |
| Total crop | 61.0 |  |  |  |  |  |  |  |  |  |  |
| AL |  | 1487.4 | 1.00 | 0.99-1.01 | 0.45 | 0.56 | 1391.1 | 1.00 | 0.99-1.01 | 0.46 | 0.57 |
| ALL |  | 1230.1 | 1.00 | 1.00-1.01 | 0.76 | 0.55 | 1155.3 | 1.00 | 0.99-1.01 | 0.85 | 0.63 |
| Viticulture | 24.7 |  |  |  |  |  |  |  |  |  |  |
| AL |  | 139.5 | 1.01 | 0.98-1.04 | 0.91 | 0.24 | 124.5 | 1.01 | 0.98-1.05 | 0.61 | 0.2 |
| ALL |  | 115.4 | 1.01 | 0.98-1.05 | 0.97 | 0.23 | 103.4 | 1.01 | 0.97-1.05 | 0.81 | 0.26 |
| Arboriculture | 13.1 |  |  |  |  |  |  |  |  |  |  |
| AL |  | 55.1 | 0.88 | 0.79-0.98 | 0.48 | 0.99 | 31.3 | 0.81 | 0.69-0.95 | 0.59 | 0.99 |
| ALL |  | 45.5 | 0.90 | 0.80-1.01 | 0.50 | 0.96 | 26.0 | 0.83 | 0.70-0.99 | 0.61 | 0.98 |
| Straw cereals | 23.8 |  |  |  |  |  |  |  |  |  |  |
| AL |  | 890.4 | 1.00 | 0.98-1.02 | 0.86 | 0.47 | 789.1 | 1.00 | 0.98-1.02 | 0.55 | 0.48 |
| ALL |  | 736.3 | 1.00 | 0.98-1.02 | 0.69 | 0.56 | 655.3 | 1.00 | 0.97-1.02 | 0.69 | 0.66 |
| Maize | 14.4 |  |  |  |  |  |  |  |  |  |  |
| AL |  | 344.6 | 0.99 | 0.95-1.04 | 0.44 | 0.6 | 325.1 | 0.99 | 0.94-1.04 | 0.59 | 0.69 |
| ALL |  | 284.8 | 0.99 | 0.95-1.04 | 0.83 | 0.60 | 270.0 | 0.99 | 0.94-1.04 | 0.91 | 0.64 |
| Rapeseed | 11.0 |  |  |  |  |  |  |  |  |  |  |
| AL |  | 143.1 | 1.01 | 0.94-1.09 | 0.72 | 0.39 | 139.8 | 1.01 | 0.93-1.10 | 0.76 | 0.38 |
| ALL |  | 118.3 | 1.02 | 0.94-1.11 | 0.66 | 0.34 | 116.1 | 1.01 | 0.92-1.10 | 0.72 | 0.43 |
| Sunflower | 11.90 |  |  |  |  |  |  |  |  |  |  |
| AL |  | 89.8 | 1.00 | 0.91-1.09 | 0.57 | 0.52 | 79.6 | 1.00 | 0.91-1.10 | 0.51 | 0.49 |
| ALL |  | 74.2 | 1.01 | 0.91-1.11 | 0.73 | 0.44 | 66.1 | 1.00 | 0.90-1.11 | 0.70 | 0.49 |
| Potatoes | 12.6 |  |  |  |  |  |  |  |  |  |  |
| AL |  | 41.3 | 1.06 | 0.94-1.20 | 0.93 | 0.15 | 31.9 | 1.09 | 0.95-1.24 | 0.83 | 0.11 |
| ALL |  | 34.1 | 1.03 | 0.90-1.18 | 0.78 | 0.31 | 26.5 | 1.04 | 0.89-1.22 | 0.69 | 0.31 |
| Fresh vegetables | 11.5 |  |  |  |  |  |  |  |  |  |  |
| AL |  | 53.2 | 1.07 | 0.97-1.17 | 0.10 | 0.09 | 38.0 | 1.05 | 0.94-1.17 | 0.29 | 0.19 |
| ALL |  | 44.0 | 1.03 | 0.92-1.15 | 0.32 | 0.29 | 31.6 | 1.00 | 0.88-1.13 | 0.53 | 0.52 |
| Dry vegetables | 9.80 |  |  |  |  |  |  |  |  |  |  |
| AL |  | 67.5 | 0.94 | 0.84-1.06 | 0.66 | 0.82 | 61.6 | 0.95 | 0.83-1.09 | 0.34 | 0.76 |
| ALL |  | 55.8 | 0.89 | 0.78-1.03 | 0.53 | 0.94 | 51.1 | 0.89 | 0.76-1.04 | 0.42 | 0.93 |
| Beet | 13.00 |  |  |  |  |  |  |  |  |  |  |
| AL |  | 95.5 | 0.94 | 0.86-1.02 | 0.73 | 0.92 | 89.0 | 0.95 | 0.87-1.04 | 0.77 | 0.86 |
| ALL |  | 78.9 | 0.93 | 0.84-1.02 | 0.94 | 0.94 | 73.9 | 0.93 | 0.84-1.03 | 0.95 | 0.91 |

^a^ The total crop density and the specific crop density in a municipality were defined as the ratio of the total area used for agriculture and the area used for the specific crop, respectively, over the total area of the municipality (based on national agricultural census data). Separate models were used for each specific crop as well as for total crops.

Q3: 3^rd^ population-weighted quartile of the crop density distribution in municipalities with total crop density≥5% or specific crop density ≥5%; E (Q3): Expected number of cases in municipalities with crop density >Q3; SIRR: Relative Standardized Incidence Ratio: multiplicative variation in the SIR for a 10% increase in the crop density derived from a linear Poisson regression model. 95%CI: 95% confidence interval; AL: Acute leukaemia; ALL: Acute lymphoblastic leukaemia;

p_loglin_: p-value of the test of departure from log-linearity hypothesis

p_slope_: p-value test for the slope parameter in the linear Poisson regression model, H0: β≤ 0 vs H1: β>0)
