# Supplementary material for: Residential proximity to croplands at birth and childhood leukaemia
Source: Environ Health. 2022 Oct 27;21:103. doi: 10.1186/s12940-022-00909-0 (PMC9615229; doi:10.1186/s12940-022-00909-0)
Supplement: Supplementary file 6 — Additional file 6: Additional Table 4. Association between ALL and BCP-ALL incidence rates and crop densitiesa in the municipalities of residence at birth, stratified by age group (mainland France, RNCE, 1990–2015). [file 12940_2022_909_MOESM6_ESM.docx]

Additional Table 4: Association between ALL and BCP-ALL incidence rates and crop densities^a^ in the municipalities of residence at birth, stratified by age group (mainland France, RNCE, 1990–2015).

|  |  | ALL (N_0-6 years_= 5 353; N_7-14years_= 1 883) | | | | |  | BCP-ALL (N_0-6 years_ = 5 201; _7-14years_ = 1 769) | | | | |
| --- | --- | --- | --- | --- | --- | --- | --- | --- | --- | --- | --- | --- |
|  | Q3 | E(Q3) | SIRR | 95% CI | p_loglin_ | p_slope_ |  | E(Q3) | SIRR | 95% CI | p_loglin_ | p_slope_ |
| Total crops | 61.0 |  |  |  |  |  |  |  |  |  |  |  |
| 0-6 years old |  | 894.1 | 1.00 | 0.99-1.01 | 0.67 | 0.52 |  | 868.7 | 1.00 | 0.99-1.01 | 0.61 | 0.55 |
| 7-14 years old |  | 318.2 | 1.00 | 0.99-1.02 | 0.71 | 0.34 |  | 299.0 | 1.00 | 0.99-1.02 | 0.77 | 0.45 |
| Viticulture | 24.7 |  |  |  |  |  |  |  |  |  |  |  |
| 0-6 years old |  | 82.7 | 1.01 | 0.97-1.05 | 0.98 | 0.33 |  | 80.3 | 1.01 | 0.97-1.05 | 0.99 | 0.27 |
| 7-14 years old |  | 30.2 | 1.02 | 0.96-1.09 | 0.91 | 0.23 |  | 28.4 | 1.02 | 0.95-1.09 | 0.94 | 0.28 |
| Arboriculture | 13.1 |  |  |  |  |  |  |  |  |  |  |  |
| 0-6 years old |  | 33.7 | 0.91 | 0.80-1.03 | 0.66 | 0.92 |  | 32.8 | 0.90 | 0.79-1.03 | 0.43 | 0.93 |
| 7-14 years old |  | 13.1 | 0.83 | 0.66-1.05 | 0.56 | 0.94 |  | 12.3 | 0.82 | 0.65-1.05 | 0.71 | 0.94 |
| Straw cereals | 23.8 |  |  |  |  |  |  |  |  |  |  |  |
| 0-6 years old |  | 560.7 | 0.99 | 0.97-1.02 | 0.46 | 0.70 |  | 544.8 | 0.99 | 0.97-1.02 | 0.43 | 0.71 |
| 7-14 years old |  | 191.4 | 1.02 | 0.99-1.05 | 0.95 | 0.09 |  | 179.8 | 1.02 | 0.99-1.05 | 0.95 | 0.13 |
| Maize | 14.4 |  |  |  |  |  |  |  |  |  |  |  |
| 0-6 years old |  | 221.9 | 1.00 | 0.94-1.05 | 0.97 | 0.56 |  | 215.7 | 0.99 | 0.94-1.05 | 0.97 | 0.63 |
| 7-14 years old |  | 57.0 | 0.98 | 0.88-1.10 | 0.40 | 0.62 |  | 53.5 | 0.98 | 0.88-1.09 | 0.56 | 0.65 |
| Rapeseed | 11.0 |  |  |  |  |  |  |  |  |  |  |  |
| 0-6 years old |  | 94.7 | 0.95 | 0.86-1.04 | 0.38 | 0.87 |  | 92.1 | 0.95 | 0.86-1.05 | 0.42 | 0.86 |
| 7-14 years old |  | 25.2 | 1.29 | 1.12-1.49 | 0.31 | <0.01 |  | 23.6 | 1.27 | 1.09-1.47 | 0.44 | <0.01 |
| Sunflowers | 11.9 |  |  |  |  |  |  |  |  |  |  |  |
| 0-6 years old |  | 53.1 | 0.99 | 0.88-1.11 | 0.70 | 0.59 |  | 51.6 | 0.99 | 0.88-1.11 | 0.67 | 0.56 |
| 7-14 years old |  | 19.5 | 1.07 | 0.89-1.28 | 0.50 | 0.23 |  | 18.4 | 1.06 | 0.88-1.29 | 0.35 | 0.25 |
| Potatoes | 12.6 |  |  |  |  |  |  |  |  |  |  |  |
| 0-6 years old |  | 24.3 | 1.05 | 0.90-1.23 | 0.84 | 0.26 |  | 23.6 | 1.06 | 0.91-1.24 | 0.78 | 0.23 |
| 7-14 years old |  | 9.1 | 1.00 | 0.76-1.31 | 0.88 | 0.51 |  | 8.6 | 1.01 | 0.76-1.33 | 0.96 | 0.47 |
| Fresh vegetables | 11.5 |  |  |  |  |  |  |  |  |  |  |  |
| 0-6 years old |  | 34.5 | 1.09 | 0.98-1.22 | 0.26 | 0.06 |  | 33.5 | 1.08 | 0.96-1.21 | 0.29 | 0.10 |
| 7-14 years old |  | 13.1 | 0.90 | 0.71-1.13 | 0.90 | 0.18 |  | 12.3 | 0.89 | 0.71-1.13 | 0.90 | 0.82 |
| Dry vegetables | 9.8 |  |  |  |  |  |  |  |  |  |  |  |
| 0-6 years old |  | 39.1 | 0.87 | 0.74-1.03 | 0.60 | 0.95 |  | 38.0 | 0.86 | 0.73-1.02 | 0.49 | 0.96 |
| 7-14 years old |  | 15.7 | 0.94 | 0.74-1.20 | 0.44 | 0.68 |  | 14.7 | 0.94 | 0.73-1.21 | 0.41 | 0.68 |
| Beet | 13.0 |  |  |  |  |  |  |  |  |  |  |  |
| 0-6 years old |  | 61.1 | 0.94 | 0.84-1.04 | 0.57 | 0.88 |  | 59.4 | 0.94 | 0.84-1.04 | 0.60 | 0.88 |
| 7-14 years old |  | 22.6 | 0.95 | 0.80-1.13 | 0.69 | 0.71 |  | 21.2 | 0.95 | 0.80-1.14 | 0.87 | 0.69 |

^a^ The total crop density and the specific crop density in a municipality are defined as the ratio of the total area used for agriculture and the area used for the specific crop, respectively, over the total area of the municipality (based on national agricultural census data). Separate models were used for each specific crop as well as for total crops.

N_0-6years_: number of cases aged 0-6 years; N_7-14years_: number of cases aged 7-14 years; Q3: 3^rd^ population-weighted quartile of the crop density distribution in the municipalities; E(Q3): Expected number of cases in the last category of crop density; SIRR: Relative Standardized Incidence Ratio: multiplicative variation in the SIR for a 10% increase in the crop density derived from a linear Poisson regression model adjusted to the observations. 95%CI: 95% confidence interval; AL: Acute leukaemia; ALL: Acute lymphoblastic leukaemia; BCP-ALL: B-cell precursor ALL;

p_loglin_: p-value of the test of departure from log-linearity hypothesis

p_slope_: p-value test for the slope parameter in the linear Poisson regression model, H0: β≤ 0 vs H1: β>0)
