# Supplementary material for: Residential proximity to croplands at birth and childhood leukaemia
Source: Environ Health. 2022 Oct 27;21:103. doi: 10.1186/s12940-022-00909-0 (PMC9615229; doi:10.1186/s12940-022-00909-0)
Supplement: Supplementary file 7 — Additional file 7: Additional Table 5. Bibliography. [file 12940_2022_909_MOESM7_ESM.docx]

Additional Table 5: Bibliography

| reference  country | Study design (period)  number of cases/controls | Materials | Crops considered and exposure assessment | Statistical analyses | Main results |
| --- | --- | --- | --- | --- | --- |
| Residence at birth |  |  |  |  |  |
| Nguyen et al. 2021  USA (California) | Case control study (1988-2008)   - 1. year old children   5788 AL cases (4721 ALL, 852 AML) and 5788 matched controls | -California Birth Registry (Random selection of controls matched to cases on date of birth and gender)  -California Cancer Registry  -California Department of Food and Agriculture  -Pesticide Uses Reporting  -Google Earth aerial satellite images  -Geocoded addresses of plant nurseries  - Distance between geocoded address at birth and plant nurseries | Crops: plant nurseries  Exposure indicator:  **distance from address at birth to plant nursery (m):** categorized in two ways  1) for all subjects within 600 m 0–75, 75–150, 150–300, and ⩾300 m (reference)  2)for all subjects within 2000m  -0–500, 500–1000, 1000–1500 and ⩾1500 m (reference)  -0–1000 and ⩾1000 m (reference) | Logistic regression model adjusted for age at diagnosis, gender, socioeconomic status, and race/ethnicity | 1) **distance to nursery ≤ 600 m**  - 0-75m *vs.* ⩾300m  AL: OR= 2.40 (0.99-5.82)  ALL: OR= 3.09 (1.14- 8.34)  2) **distance to nursery ≤ 2000 m**  -1000-1500m *vs.* ⩾1500m  AL: OR= 1.05 (0.88- 1.25)  ALL: OR=1.09 (0.90-1.33)  -500-1000m *vs.* ⩾1500m  AL: OR= 1.01 (0.85- 1.19)  ALL: OR=0.98 (0.81-1.18)  -0-500m *vs.* ⩾1500m  AL: OR= 0.99 (0.82-1.19)  ALL: OR= 1.00 (0.82-1.24)  -0-1000 *vs.* ⩾1000m  AL: OR= 1.02 0.94-1.10)  ALL: OR= 1.03 (0.94-1.12) |
| Patel et al. 2020  Denmark | Case cohort (1996-2003)  0-14 year old children  9,394 births of which 61 AL cases | -Danish National birth cohort (random sample of the live births ~10%)  -Danish cancer registry  -General registry of agriculture  -Pesticide sales data  -Address at birth geocoding with construction of several buffers of different size (250m,500m,1000m,2000m) | Crops: winter cereals, spring cereals, grass/clover,  winter rapeseed, peas, maize, sugar beet, and seed crops  Exposure indicator:  1) **agricultural area (ha)** for each month during pregnancy by planting and harvesting periods of the various crops: categorized in two ways a) by tertile of the crop distribution: for total area and common crops  b) any crop *vs.* no crops for less common crops  2)**pesticide use** based on sales data | Cox model to estimate Hazard ratio adjusted for gender, maternal age, farm animals within 1000m | 1) **Agricultural area**  - Total agricultural area (highest tertile (>24ha) *vs.* no crops within 500m buffer):  HR=2.0 (1.0-3.8)  - Grass/clover (Highest tertile (>1.1ha) *vs.* no crops):  HR = 3.1 (1.2-7.7)  - Peas (>0 ha *vs.* 0):  HR= 2.4 (1.02-5.4)  **-**Maize (>0 ha *vs.* 0):  HR=2.8 (1.1-6.9)  **2)Pesticides**: no association with childhood leukaemia |
| Carozza et al. 2009  USA (Texas) | Case-control (1990-1998)  Children 0-8 years old  1,778 cancer cases (606 AL, 464 ALL) and 1,802 matched controls | -Birth registry (random selection of controls matched to cases on birth date and gender)  -Texas Cancer registry  -Aerial photographs (1995-1997) for field identification  -Birth address geocoding with construction of 1000 m buffer | Crops: total agricultural area  Exposure indicators:  1) **FMI (Field Mass index)** within buffer = agricultural area / (distance to fields from residence)^2^: reference = 0; low<0.54; high≥0.54)  2) **Mean of total agricultural area** within buffer: low (0.42–12,5), medium (12,5–37,2), high (37,2–981,09)  3) **Mean of distance (m)** between residence and fields within buffer: low (175–802); medium (803–932); high (933–1000). | -Unconditional logistic regression adjusted for race, gender, date of birth  **FMI** : OR_1_=low vs. ref; OR_2_=high vs. ref  **Mean of total agricultural area:** OR_1_=low ; OR_2_= medium; OR_3_=high  **Mean of distance to fields:** OR_1_=low ; OR_2_= medium; OR_3_=high | No association between crops and childhood leukaemia regardless of the exposure indicator used  -Results for **FMI**:  AL: OR_1_= 0.9 (0.6-1.2) ; OR_2_= 1.0 (0.7-1.4)  ALL: OR_1_= 0.9 (0.6-1.3) ; OR_2_= 1.1 (0.8-1.5)  -Result for **mean of total agricultural area**  AL: OR1= 0.8 (0.5-1.1) ; OR_2_= 0.8 (0.5-1.2); OR_3_= 1.3 (0.0-1.9)  **ALL**: OR_1_= 0.9 (0.6-1.3) ; OR_2_= 0.8 (0.5-1.0); OR_3_= 1.4 (0.9-2.1)  -Results for **mean of** **distance to fields**:  **AL**: OR1= 0.9 (0.6-1.4) ; OR_2_= 1.2 (0.8-1.8); OR_3_= 0.7 (0.5-1.1)  ALL: OR_1_= 1.0 (0.6-1.5) ; OR_2_= 1.2 (0.8-1.9); OR_3_= 0.8 (0.5-1.2) |
| Thompson et al. 2008  USA (Texas) | Ecological study (1990-2003)  Children 0-13 years old  1,248 AL cases* (968 ALL  180 AML) | -Birth registry  -Texas cancer registry | Major crops: maize, soya beans,  wheat, and sorghum  Exposure indicator  - **Percent of county area dedicated to major crops (combined):** categorised as extensive cropping (≥20%) *vs.* low cropping (≤20%) | Bayesian Poisson regression models including hazardous air pollutants and population density: estimate of SMR for >20% vs. ≤20% | ALL : SMR=1.01 (0.79-1.28)  AML: SMR=0.75 (0.41-1.27) |
| Walker et al. 2007  USA (Texas) | Case-control (1986-1998)  Children 0-14 years old  3,487 cancer cases (1,178 AL, 927 ALL, 186 AML) and 3,487 matched controls | -Birth registry (random selection of controls matched to cases on birth date and gender)  -Texas cancer registry  -Agricultural census 1987,1992,1997  -Pesticide use data from the National Agricultural Statistics Service (NASS) Agricultural Chemical Use database 1990-1997 | Crops : total agricultural area (cotton+ wheat+ sorghum+ maize+ soya beans)  exposure indicators:  1) **Percent of total agricultural area** in relation to the total area of county of residence at birth (categorised as <25%, 25-49.9%, ≥50%)  2) **total county-specific pesticide exposure** index: low (≤1.31), medium (]1.31-5]), high(>5) | Unconditional logistic regression: OR_1_=25%-49.9% *vs.* <25%; OR_2_= ≥50% *vs.* < 25% | 1)**Total agricultural area**  No association between percent of total agricultural area in county and childhood leukaemia  AL : OR_1_=0.9(0.8-1.1); OR_2_=0.9(0.7-1.3)  ALL: OR_1_=0.9(0.8-1.1); OR_2_=1.0(0.7-1.4)  AML: OR_1_=1.0(0.7-1.4); OR_2_=1.0(0.5-1.9)  2) **Total county-specific pesticide exposure**  No association between total county-specific pesticide exposure and childhood leukaemia |
| Residence at diagnosis | |  |  |  |  |
| Coste et al. 2020  France | Ecological study (1990-2014)  Children 0-14 years old  11,487 AL cases (9,488 ALL and 1,803 AML) | -French national cancer registry  -Population census data  - Agricultural census data 1988, 2000 and 2010 | Exposure indicator:  -**Crop densities** (total agricultural and specific crop areas/total area of municipality): considered as continuous variable or categorized as population weighted quartiles to build qualitative variable  a) for total agricultural area: <5%; 5%-Q1; Q1-Q2; Q2-Q3; ≥Q3  b) for specific crops: total agricultural density>5%; total agricultural density ≥5% + specific crop density<5%; 5%-Q1; Q1-Q2; Q2-Q3; ≥Q3 | Poisson regression  -qualitative model: SIR for each category of crop density  - quantitative model: SIRR for 10% increase in crop density | **Total agricultural density**  -Qualitative variable (SIRs for highest quartile ≥63%)  AL: SIR= 0.99 (0.94–1.03)  ALL: SIR= 0.98 (0.93–1.03)  AML: SIR= 0.99 (0.89–1.11)  -Continuous variable (SIRR for 10% increase in crop density)  AL: SIRR= 1.00 (0.99–1.01)  ALL: SIRR= 1.00 (0.99–1.01)  AML: SIRR= 0.99 (0.98–1.01)  **Viticulture**  -Qualitative variable (SIRs for highest quartile ≥25%)  AL: SIR= 1.16 (1.02–1.32)  ALL: SIR= 1.17 1.01–1.35  -Continuous variable (SIRR for 10% increase in viticulture density)  AL: SIRR= 1.03 (1.00–1.06)  ALL: SIRR=1.04 (1.01–1.07)  -No association between other types of crops and childhood AL |
| Malagoli et al. 2016  Italy (Modena and the Reggio Emilia provinces) | Case-control (1998-2011)  Children 0-14 years old  111 AL cases and 444 matched controls | -Italian hospital-based registry of childhood malignancies  -population data from the National Health Services Local Health Units of Modena and Reggio Emilia (random selection of controls matched to cases on year of birth, gender, province and calendar year of residence)  -Land use Map 2003  -Address at diagnosis/inclusion geocoding with construction of 100-m circular buffers | Crops: total agricultural land, arable crops (wheat, maize, barley), orchards,  vineyards and vegetables  Exposure indicator:  **Percent of total agricultural area and % of specific crop area in the buffer**: categorized to build 2 variables :  a)1^st^ variable: reference=0; <median; ≥median  b)2^nd^ variable: ≥95% *vs.* <95% (just for total agricultural land and arable crops); | Conditional logistic regression adjusted for outdoor benzene, PM_10_, magnetic fields from high-voltage power lines:  -1^st^ variable: OR_1_= <median *vs.* 0; OR_2_= ≥median *vs.* 0  -2^nd^ variable : OR= ≥95% *vs.* <95% | **Total agricultural area**  a)1^st^ variable  AL: OR_1_= 0.85 (0.44-1.63) ;  OR_2_= 0.71 (0.36-1.41)  b) 2^nd^ variable  AL: OR= 0.64 (0.18–2.22) ;  (<5years) OR= 1.18 (0.24-5.82)  ALL: OR= 0.55 (0.12–2.46)  AML: OR= 1.00 (0.09–11.64)  **Arable crops**  a)1^st^ variable  AL: OR_1_= 0.76 (0.39-1.49) ;  OR_2_= 0.61 (0.30-1.23)  b) 2^nd^ variable  AL: OR= 2.04 (0.50-8.35)  ALL: OR= 2.14 (0.38-11.93)  AML: OR= 1.84 (0.14-24.24)  No association between AL and other specific crops |
| Gomez-Barroso et al. 2016  Spain | Case-control (1996-2011)  Children 0-14 years old  1,062 AL cases and 6,451 matched controls | -Spanish Registry of Childhood Tumours (RETI-SEHOP)  -Birth Registry of the National Statistics Institute (INE) (random selection of controls matched to cases on year of birth, gender and region of residence)  -Corine Land Cover 2006  -Industrial database for industrial pollutants  -Address at diagnosis for the cases and address at birth for controls; geocoding with construction of 1000-m buffers | Crops: arable land or permanently irrigated land (Irrigated); rice; vineyards; fruit; olives; and heterogeneous agricultural areas, including annual crops associated with permanent crops  Exposure indicator:  - **global crop index** and **crop index for specific crops** (percent of crop area within 1 km buffers): categorized as 0 (not exposed), 1^st^, 2^nd^, 3^rd^ and 4^th^ quartiles | Mixed multiple unconditional logistic regression models including the region as random effect :  - qualitative model: OR_1_=1^st^ quartile *vs.* 0; OR_2_=2^nd^ quartile *vs.* 0; OR_3_=3^rd^ quartile *vs.* 0; OR_4_=4^th^ quartile *vs.* 0  - linear model: OR for 1% increase in crop densities | **North region**  1) Qualitative model: higher risk of total AL in all categories for almost all the crops considered  2) Linear model  -Total agricultural land: OR=1.01 (1.01, 1.02);  -Irrigated land: OR=1.01 (1.01, 1.02)  -Heterogeneous crops: OR= 1.01 (1.01, 1.02)  -Fruit: OR=1.00 (0.99, 1.02)  -Vineyards: OR= 1.00 (0.99, 1.02)  -Olives: OR= 1.02 (0.99, 1.05)  **Madrid region**  1) Qualitative model  -Total agricultural land: OR_1_= 0.37 (0.05, 2.71),OR_2_= 2.64 (1.42, 4.91), OR_3_= 2.40 (1.18, 4.88),OR_3_= 3.91 (1.68, 9.08)  2) Linear model  -Total agricultural land: OR= 1.03 (1.02, 1.05)  -Irrigated land: OR= 1.02 (0.99, 1.05)  -Heterogeneous crops: OR= 1.05 1.03, 1.08) |
| Booth et al. 2015  USA (6 Midwestern states: Illinois,  Indiana, Iowa Michigan, Missouri and Ohio) | Ecological study (2004-2008)  Children 0-4 years old  664 AL cases (518 ALL and 94 AML cases) | -Cancer registries and Surveillance, Epidemiology,  and End-Results (SEER) program (for Iowa)  -Population census data  -Agricultural census for year 2000 and 2007 | Crops: total agricultural land, barley, dry beans, maize, hay, oats, sorghum, soya beans, sugar beet, and wheat  Exposure indicator:  - **Percent of agricultural area** (total and specific crop area) in relation to county total area considered as a continuous variable or categorized as:  a)1^st^ indicator : ≤median *vs.* >median;  b) 2^nd^ indicator: based on quartiles (equal number of counties in each group)  c) 3^rd^ indicator: 0 (reference), ≤median, >median (for barley, dry beans, oats, sorghum, and sugar beet) | -GEE Poisson regression model adjusted for year, race, gender, county median household income, educational attainment and population density  -Analyses only of counties with population less than 300,000 (551 counties) | **Result for qualitative variable** (RR for density >Q4)  -Total agricultural land (Q4=73.9%)  AL: RR= 1.01 (0.77-1.31)  ALL : RR= 1.05 (0.78-1.42)  AML : RR= 0.64 (0.27-1.53)  **Results for continuous variable** (RR for 1% increase in crop density)  -Dry beans  AL: RR= 1.09 (1.03–1.14)  ALL: RR=1.10 (1.04, 1.16)  AML: RR= 0.94 (0.75, 1.17)  -Sugar beet  AL: RR = 1.11 (1.04, 1.19)  ALL: 1.11 (1.02, 1.21)  AML: 1.01 (0.78, 1.30)  -Oats  AL: RR= 0.89 (0.64, 1.22)  ALL: RR= 0.66 (0.44, 0.98)  AML: RR= 2.03 (1.25-3.28) |
| Carozza et al. 2008  USA | Ecological study (1995-2001)  children 0-14 years old  6,168 AL cases* | -Cancer registries  -Population census data  -Agricultural census 1997 | Crops: barley, maize, cotton, oats, soya beans and wheat  Exposure indicator:  -**Percent of agricultural area** in relation to county total area categorized as:  a) total agricultural land: reference: <20%, medium: [20-60[, high: ≥60%  b) specific crop:  not exposed: specific crop density=0% and total agricultural area density <20% (ref), exposed: specific crop density >0% | Logistic regression adjusted for age and gender :  -Total agricultural densities: OR_1_=medium *vs.* <20%; OR_2_= high *vs.* <20%  -Specific crop densities: OR= exposed *vs.* not exposed | **Total agricultural land**  AL: OR_1_=1.0 (0.9-1.1); OR_2_=1.2 (1.1–1.3)  ALL: OR_1_=1.0 (0.9-1.1); OR_2_=1.3 (1.1–1.4)  AML: OR_1_=1.2 (1.1-1.4); OR_2_ =1.8 (1.4–2.3)  **Maize**  AML: OR=1.2 (1.0–1.5);  **Oats**  ALL: OR=1.2 (1.0-1.4)  **Soya beans**  AML: OR=1.4 (1.1–1.7) |

*Calculated based on number of cases indicated in tables

SIR= Standardized Incidence Ratio; SIRR= Standardized Incidence Rate Ratio; SMR= Standardized Morbidity Ratio;
